# Bridging physical intuition and hardware efficiency for correlated electronic states: the local unitary cluster Jastrow ansatz for electronic structure†

**DOI:** 10.1039/d3sc02516k

**Published:** 2023-09-21

**Authors:** Mario Motta, Kevin J. Sung, K. Birgitta Whaley, Martin Head-Gordon, James Shee

**Affiliations:** a IBM Quantum, IBM Research – Almaden San Jose CA 95120 USA mario.motta@ibm.com; b IBM Quantum, IBM T. J. Watson Research Center Yorktown Heights NY 10598 USA; c Department of Chemistry, University of California Berkeley CA 94720 USA; d Berkeley Quantum Information and Computation Center, University of California Berkeley CA 94720 USA; e Challenge Institute for Quantum Computation, University of California Berkeley CA 94720 USA; f Chemical Sciences Division, Lawrence Berkeley National Laboratory Berkeley CA 94720 USA; g Department of Chemistry, Rice University Houston TX 77005 USA james.shee@rice.edu

## Abstract

A prominent goal in quantum chemistry is to solve the molecular electronic structure problem for ground state energy with high accuracy. While classical quantum chemistry is a relatively mature field, the accurate and scalable prediction of strongly correlated states found, *e.g.*, in bond breaking and polynuclear transition metal compounds remains an open problem. Within the context of a variational quantum eigensolver, we propose a new family of ansatzes which provides a more physically appropriate description of strongly correlated electrons than a unitary coupled cluster with single and double excitations (qUCCSD), with vastly reduced quantum resource requirements. Specifically, we present a set of local approximations to the unitary cluster Jastrow wavefunction motivated by Hubbard physics. As in the case of qUCCSD, exactly computing the energy scales factorially with system size on classical computers but polynomially on quantum devices. The local unitary cluster Jastrow ansatz removes the need for SWAP gates, can be tailored to arbitrary qubit topologies (*e.g.*, square, hex, and heavy-hex), and is well-suited to take advantage of continuous sets of quantum gates recently realized on superconducting devices with tunable couplers. The proposed family of ansatzes demonstrates that hardware efficiency and physical transparency are not mutually exclusive; indeed, chemical and physical intuition regarding electron correlation can illuminate a useful path towards hardware-friendly quantum circuits.

## Abstract 2

A prominent goal in quantum chemistry is to solve the molecular electronic structure problem for ground state energy with high accuracy.

## Graphical abstract

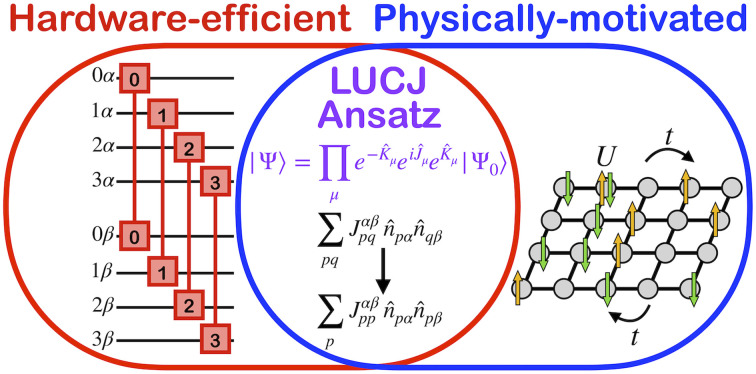

## 1 Introduction

Obtaining the ground-state energy of a correlated electronic system has been recognized as an important field of application of quantum computers.^1–6^ In recent years, various algorithms have delivered promising results in the calculation of potential energy curves, ground- and excited-state energies and correlation functions for various molecular systems.^7–10^ Some prominent examples of such algorithms include the variational quantum eigensolver (VQE),^6,11^ quantum subspace expansion (QSE),^12,13^ imaginary-time evolution,^14^ adiabatic state preparation (ASE),^15^ and quantum phase estimation (QPE).^16^ The feasibility of such algorithms on near-term quantum devices depends on the ability of approximate wavefunction ansatzes to yield accurate energies and properties while requiring the smallest possible amount of quantum resources.

The most widely used ansatz for eigenstates of electronic Hamiltonians is the unitary coupled cluster form with single and double excitations (qUCCSD):^17^

1 |*ψ*〉 = e^*T̂*−*T̂*^†^^|*Φ*_0_〉

where |*Φ*_0_〉 is a reference determinant, often the Hartree–Fock state, and 
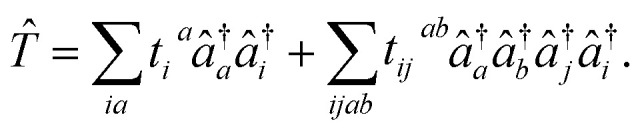
 As with classical (projective) coupled cluster methods,^18^ qUCCSD preserves several desirable properties that are advantageous for the computationally efficient modeling of chemical reactions including size consistency, size extensivity, differentiability, and invariance under occupied–occupied and virtual–virtual orbital rotations. The exponential ansatz also provides a compact, approximate description of infinite-order electronic excitations, critical for even qualitatively correct physical descriptions of, *e.g.*, many-body screening and polarization effects.^19^ While less studied relative to classical coupled cluster models, the unitary variant is better suited to describe some static correlation effects beyond two-electron problems when solved variationally.

The qUCCSD ansatz is especially suited for quantum computers, because the cost of exactly (*i.e.*, variationally) evaluating the energy *E* = 〈*Ψ*|*Ĥ*|*Ψ*〉 over a qUCCSD state *Ψ* is polynomial with system size on a quantum computer and factorial on a classical one.^20^ Despite such favorable polynomial scaling, the qUCCSD ansatz contains 
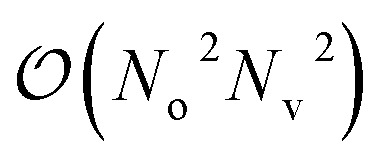
 parameters, where *N*_o_ and *N*_v_ are the numbers of occupied and virtual orbitals in a mean-field reference wavefunction, and a quantum circuit of depth 
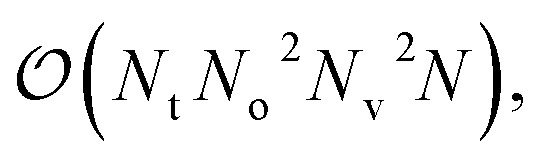
 where *N* = *N*_o_ + *N*_v_ and *N*_t_ is the number of Trotter steps used to approximately implement the qUCCSD ansatz. As a result, quantum simulations based on the qUCCSD ansatz vastly exceed the capabilities of contemporary quantum devices in the noisy intermediate-scale quantum (NISQ) era in terms of gate fidelity, qubit coherence time, and qubit connectivity.

The high computational cost of qUCCSD calculations originates from the rapidly growing number of double-excitation amplitudes. However, chemical intuition (based roughly on the locality of most electronic correlations despite growing system size) suggests that more compact ansatzes can be designed, leveraging formal properties of the cluster operator. This observation has prompted various authors to introduce approximate flavors of qUCCSD including the low-depth ansatz,^21^ the low-rank approximation,^22^ and the *k*-fold product of a unitary pair coupled cluster.^23^ Yet, further quantum resource reductions would be highly desirable.

An alternative approach to formulating a compact many-body wavefunction was introduced by Jastrow in 1955,^24^ which exponentiates a function of the relative position of two particles, *i.e.*, e^*f*(**r**_*ij*_)^, such that relatively short-ranged two-body correlation effects are efficiently captured. This wavefunction is widely used in variational Monte Carlo for continuous systems^24–26^ and lattice models^27,28^ in both first^29–32^ and second^33,34^ quantization. More recently, the Jastrow has been used in the context of quantum computation.^35,36^ The starting point for this study is the unitary cluster Jastrow ansatz,^36^ which is a unitary variant of the cluster Jastrow form introduced by Neuscamman in the context of the antisymmetrized geminal power reference state.^33,34,37^ This ansatz has been demonstrated to yield promising accuracy with reduced circuit depths in comparison to qUCCSD.^36,38^

A challenging frontier of many-body quantum mechanics involves the description of strongly correlated fermionic systems. By “strongly correlated” we refer to quantum states in which more than one Slater determinant has significant weight in the wavefunction, a situation which results from the presence of nearly degenerate eigenstates. In the chemistry community, this is also known (equivalently) as “multi-reference character,” “static” or “non-dynamic” correlation. Strong correlation is challenging for most available quantum chemical methods. For instance, it defeats non-degenerate perturbation and response theories based on a single reference configuration, can require often prohibitively high orders of cluster operators in coupled cluster approaches, and is difficult to treat in a balanced fashion along with dynamic correlation (which can be thought of as short-range, instantaneous electron–electron repulsion). Molecular systems which exhibit strong correlation in ground (and many low-lying excited) states include di-/poly-radical organic chromophores, polynuclear transition metal compounds, and organometallic complexes with redox-noninnocent ligands.^39^ Even simple molecules which are stable (closed-shell) at their equilibrium geometry can be pushed into regimes of strong correlation when bonds are stretched and before they are broken.

The unifying feature of most strongly correlated electronic states is that localized, aligned spins are recoupled through anti-ferromagnetic interactions into low-spin states of open-shell character. A catalytically relevant example includes the Cu_2_O_2_ functional unit,^40,41^ in which each Cu^2+^ d^9^ ion has a localized unpaired electron which couples to form an open-shell singlet state. A simpler example, which nevertheless preserves much of the same physics, can be found in the intermediate region along the potential energy curve of stretched H_2_, *i.e.* a diradicaloid singlet. In both cases, restricted Hartree–Fock (RHF) produces a qualitatively incorrect closed-shell electron configuration and unrestricted HF (UHF) suffers from spin contamination. To rectify this, we draw inspiration from the Hubbard Hamiltonian:2
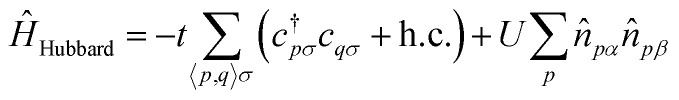
which contains a nearest-neighbor hopping term and an on-site repulsive interaction, which penalizes double-occupancy. This model has been used successfully to describe emergent effects due to strong electron correlation such as Mott insulator transitions^42–44^ and high-temperature superconductivity in cuprates.^45–48^

Herein we present a variational ansatz resembling a reference RHF state time-evolved under a Hubbard Hamiltonian. Starting from the unitary cluster Jastrow wavefunction, we keep only on-site, opposite-spin and nearest-neighbor, same-spin number–number terms. The result is a single-reference ansatz that can access open-shell character *via* a correlator which penalizes double-occupancy, and that captures strong (and weak) correlations in a systematically improvable way. The physically transparent form of the local cluster Jastrow family of ansatzes also provides a natural path to hardware efficiency: SWAP gates are no longer necessary for all qubit topologies (square, hex, and heavy-hex), and the ansatz can be implemented *via* a sequence of fSim gates that is of lower depth than that of qUCCSD, native to state-of-the-art quantum hardware utilizing tunable couplers.^49^

## 2 Methods

### 2.1 Canonical ansatz

The unitary cluster Jastrow (UCJ) ansatz has the form^36^ of *L*-fold products (or a product of *L* layers)3
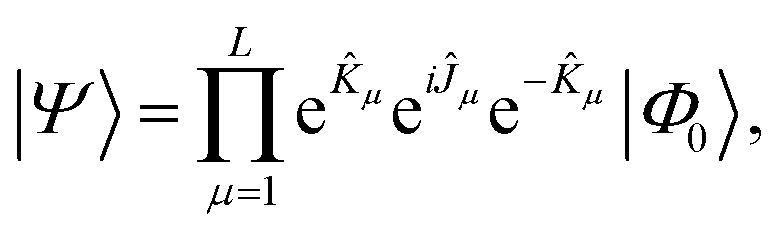
where4
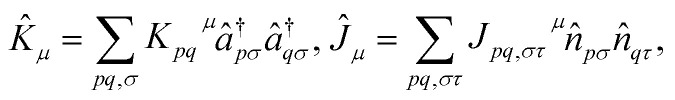
are one-body operators. In eqn (4), *p*, *q* = 0…*N* − 1 label molecular spatial orbitals and *σ*, *τ* label spin polarizations (*α*, *β* for spin-up and spin-down electrons, respectively). *K*_*pq*_^*μ*^ has complex matrix elements and is anti-Hermitian, whereas *J*_*pq*,*στ*_^*μ*^ has real matrix elements and is symmetric. The UCJ ansatz commutes with the total spin operator *S*_*z*_ if

5 *J*_*pq*,*αα*_^*μ*^ = *J*_*pq*,*ββ*_^*μ*^, *J*_*pq*,*αβ*_^*μ*^ = *J*_*pq*,*βα*_^*μ*^,

(note that commutation with the *S*^2^ operator requires *J*_*pq*,*αα*_^*μ*^ = *J*_*pq*,*αβ*_^*μ*^) leading to the expression6
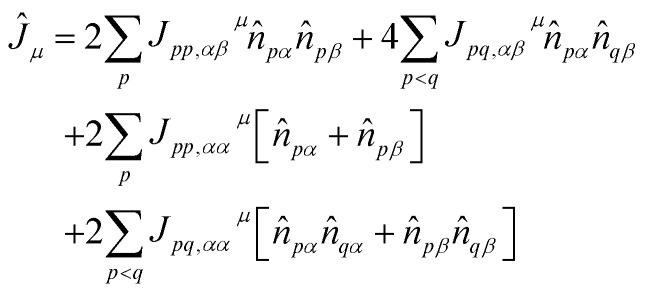
which is assumed throughout this work. The UCJ ansatz can be related to a twice-factorized low-rank decomposition of the qUCCD ansatz,^22,36^ and the *L*-product form is such that the exact full configuration interaction wavefunction can be obtained *via*eqn (3).^36,50^

Despite the many desirable properties of the UCJ ansatz, its implementation on contemporary quantum devices is challenging due to limited qubit connectivity, gate fidelity, and qubit coherence. To understand this observation, let us consider the widely used Jordan–Wigner (JW) representation of electrons in *N* spatial orbitals onto a system of 2*N* qubits,7
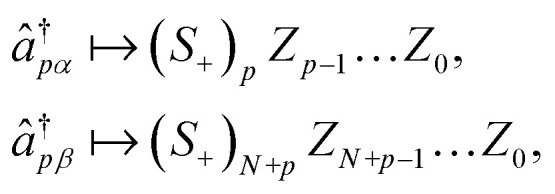
where (*S*_+_)_*p*_ = (*X*_*p*_ + *iY*_*p*_)/2 is the raising operator for qubit *p*, and *X*, *Y*, *Z* denote the 2 × 2 Pauli matrices. In the JW representation, the operator e^−*K̂*_*μ*_^ can be implemented^51^ by using a quantum circuit of 
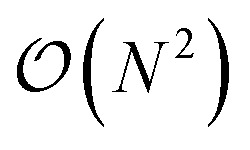
 Givens rotations acting on adjacent qubits on a device with linear connectivity, using *e.g.* the design introduced originally by Reck *et al.*,^52^ or the more economical one proposed by Clements *et al.*^53^

The operator e^*iĴ*_*μ*_^ is mapped onto a quantum circuit of the form8
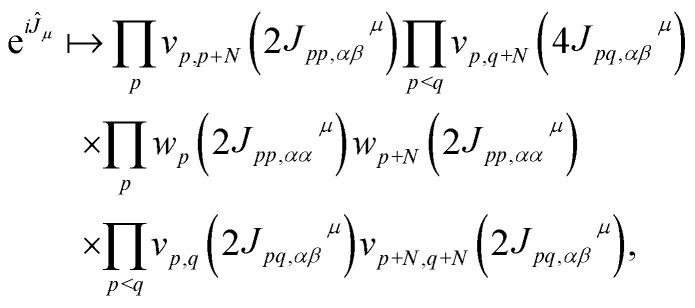
where 
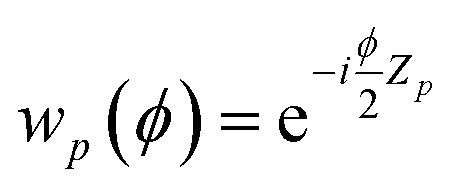
 is a single-qubit gate and9
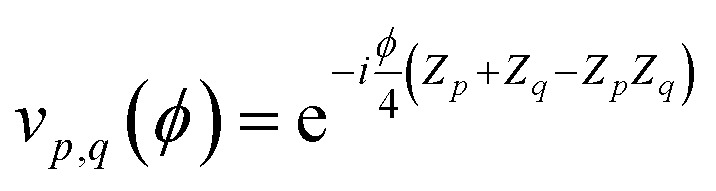
is a two-qubit gate. While the circuit in eqn (8) comprises 
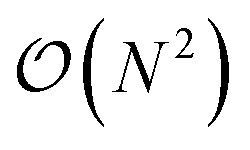
 two-qubit gates, it requires all-to-all qubit connectivity or the use of a fermionic swap network.^51^

### 2.2 Local ansatzes

**Fig. 1:**
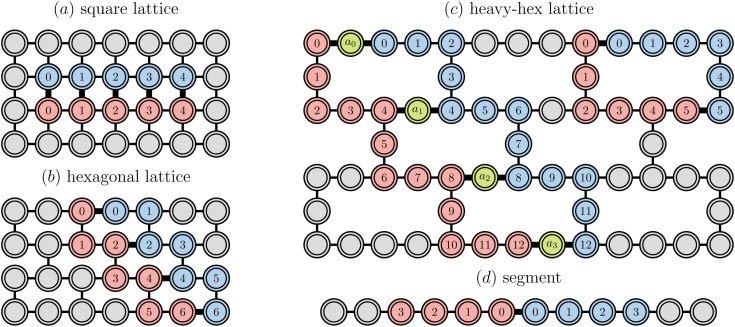
Device topologies considered in this work. Gray and colored circles indicate qubits, and black lines connect adjacent qubits in the topology of the device. Red and blue circles indicate qubits onto which, respectively, spin-up and spin-down molecular orbitals are mapped, and green circles indicate ancillae. In the case of the heavy-hex lattice (panel (c)), we consider both a “zig-zag” pattern (left) and a loop (top right). A number inside a red/blue circle denotes the index (*p* in the main text) of the spatial orbital whose occupation is encoded in the corresponding qubit, and thicker black lines connect (*p*, *α*) and (*p*, *β*) spin orbitals.

The key idea of this work is to introduce a “local” approximation of the UCJ ansatz, which makes the following modifications for opposite-spin and same-spin number–number terms:10
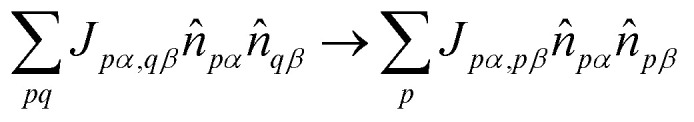
11
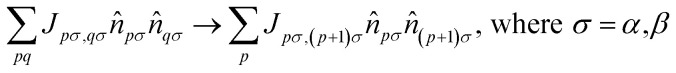
In other words, we introduce a set of sparse matrices *J*_*pα*,*qα*_^*μ*^, *J*_*pα*,*qβ*_^*μ*^ such that the quantum circuit eqn (8) only comprises gates *v*_*p*,*q*_ acting on adjacent qubits in the topology of a certain device,12
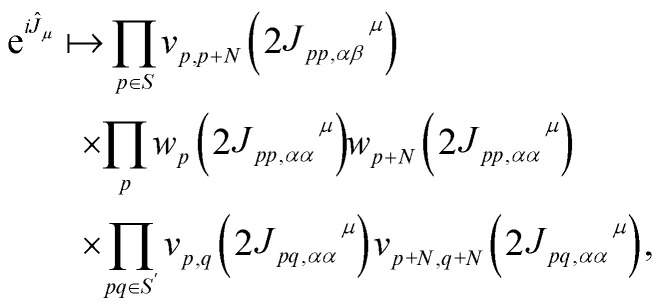
where *S* and *S*′ denote sets of qubits encoding the occupation of spin-up/down orbitals. To justify eqn (12), and define *S* and *S*′, it is useful to examine Fig. 1. It is natural to arrange qubits encoding the occupation of spin-up/down orbitals (represented by red/blue circles, respectively) along horizontal segments, so the operations e^*K̂*_*μ*_^ can be implemented acting exclusively on neighboring qubits (connected by black lines). This qubit arrangement leads naturally to the choice

13 *S*′ = {(*p*,*p* + 1), *p* = 0…*N* − 2},

*i.e.* to retain same-spin number–number terms acting on adjacent orbitals (circles of identical colors connected by black lines). Similarly, we restrict opposite-spin terms to pairs of adjacent qubits (circles of different colors connected by thick black lines). As an example, consider the device with hexagonal-lattice topology shown in Fig. 1b: for even integers *p* = 2*k*, *k* = 0…⌊(*N* − 1)/2⌋, where *N* is the number of spatial orbitals and the *p*-th qubit of the spin-up line is connected with the *p*-th qubit of the spin-down line (by thick black lines connecting qubits of different colors), so that *v*_*p*,*p*+*N*_ gates can be applied to qubits with *p* ∈ *S*_hexagonal_ = {2*k*, *k* = 0…⌊(*N* − 1)/2⌋}. Similarly, for the device topologies sketched in Fig. 1, one has14
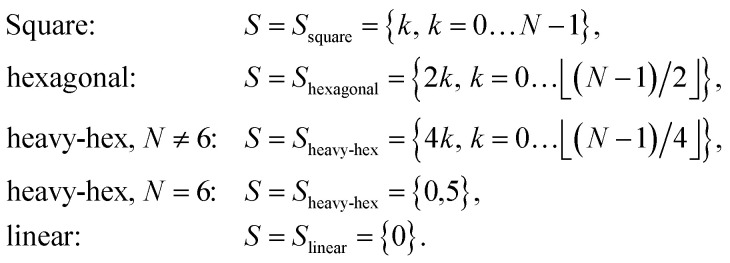


The resulting “local” ansatz will be abbreviated as LUCJ.

### 2.3 Quantum circuits

**Fig. 2:**
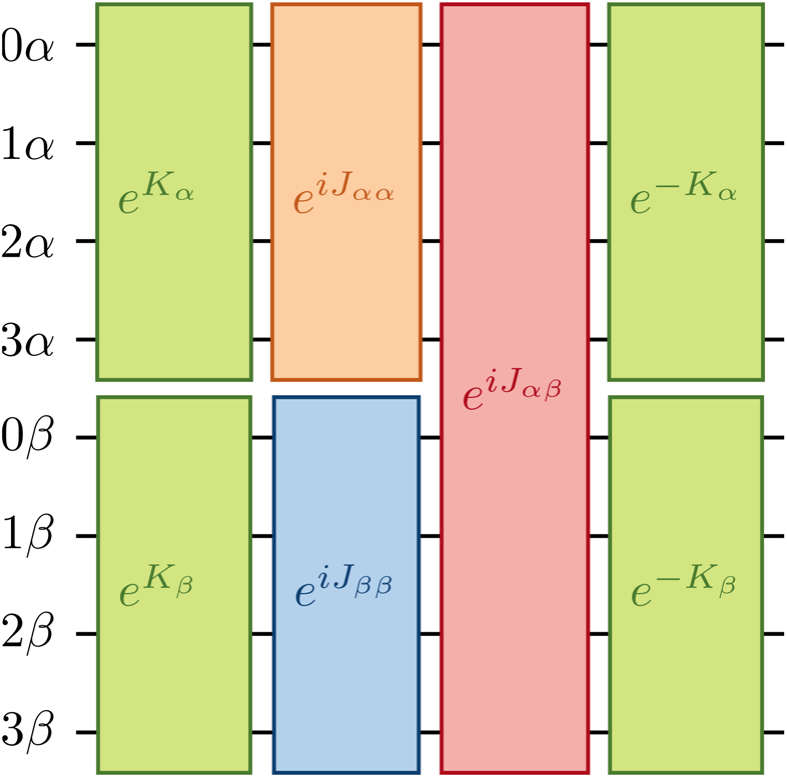
Implementation of a layer of the Jastrow ansatz using a change-of-basis unitary exp(*K*_*α*_ + *K*_*β*_) (green blocks), and a cluster operator comprising a same-spin term exp(*iJ*_*αα*_ + *iJ*_*ββ*_) (orange and blue blocks) and an opposite-spin term exp(*iJ*_*αβ*_) (red block), illustrated for *N* = 4 spatial orbitals.

Fig. 2 provides a schematic implementation of a single layer of the ansatz written in eqn (3), for the case of a four orbital problem (8 spin-orbitals).

**Fig. 3:**
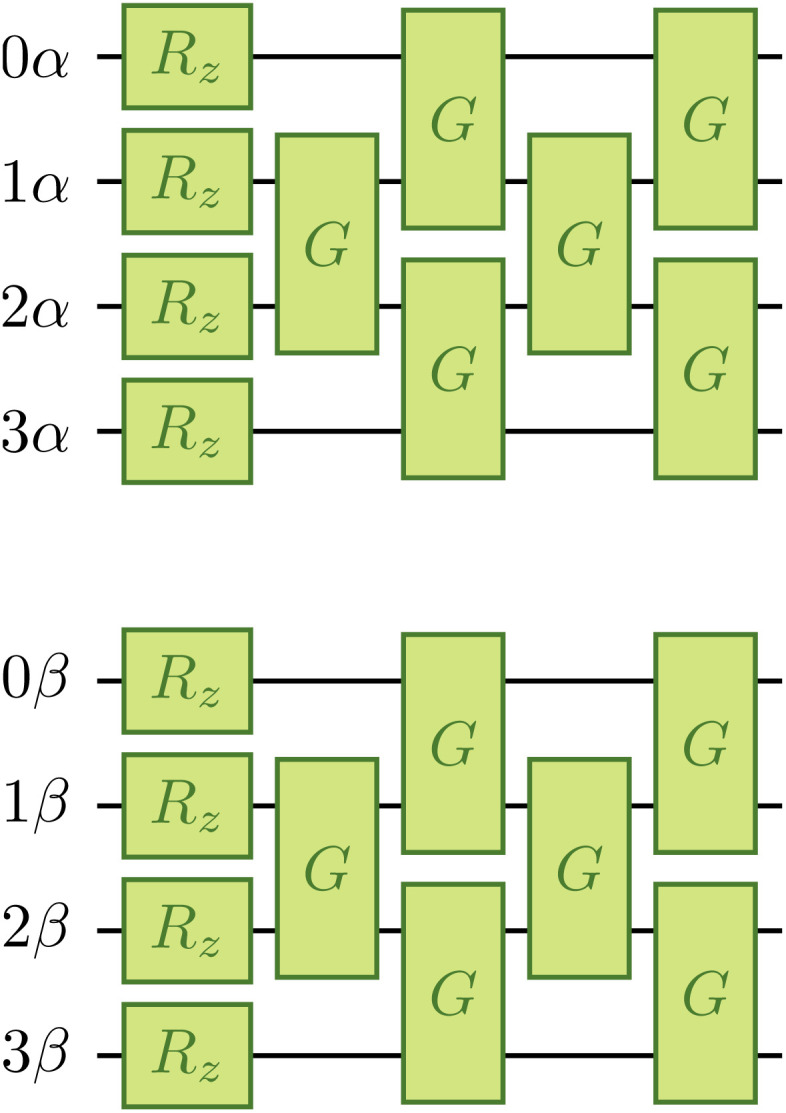
Implementation of a change of basis exp(*K*_*α*_ + *K*_*β*_) as a composition of 2*N R*_*z*_ gates and *N*(*N* − 1) *XX* + *YY* gates *G*(*θ*, *β*) arranged in *N* layers (marked as *G*, omitting parameters to avoid clutter), illustrated for a system of *N* = 4 spatial orbitals.

The one-body blocks (Fig. 2 green), which correspond to orbital rotations within a spin sector (*α* or *β*), are composed of single-qubit *R*_*z*_ gates followed by a sequence of Givens' rotations, illustrated in Fig. 3. These are unaffected by any local approximations to the UCJ wavefunction.

**Fig. 4:**
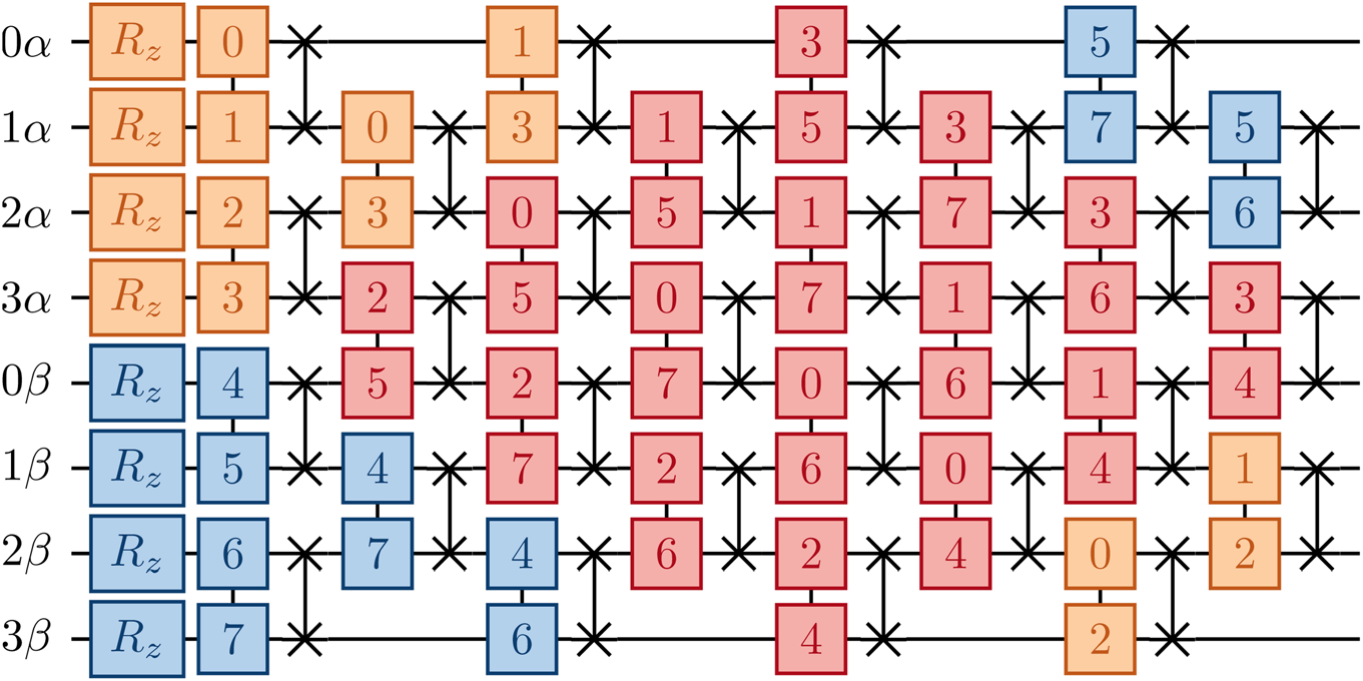
Implementation of a cluster operator exp(*iJ*_*αα*_ + *iJ*_*ββ*_ + *iJ*_*αβ*_) with all-to-all ansatz connectivity, illustrated for *N* = 4 spatial orbitals. Linear device connectivity is assumed, and a generalized SWAP network is employed so that logical operations are performed on physically adjacent qubits only. Boxes connected by a vertical line denote a number–number gate *U*_nn_(*φ*), and numbers on connected boxes denote the pair of qubits acted upon by a number–number gate, numbered from 0 to 2*N* − 1. Crosses connected by a vertical line denote a SWAP gate. Orange, blue, and red boxes denote terms of the *J*_*αα*_, *J*_*ββ*_ and *J*_*αβ*_ operators, respectively.

Currently only trapped ion architectures have all-to-all connectivity. All other devices, *e.g.* superconducting qubits, have limited qubit connectivity. As a consequence, qubits must be swapped to realize gates acting on non-adjacent spin-orbitals. For example, in Fig. 4 we show an example of how the same spin (*αα*, orange/blue) and opposite spin (*αβ*, red) components of the e^*iĴ*^ part can be optimally implemented on a device with linear connectivity using a SWAP network.^54^

**Fig. 5:**
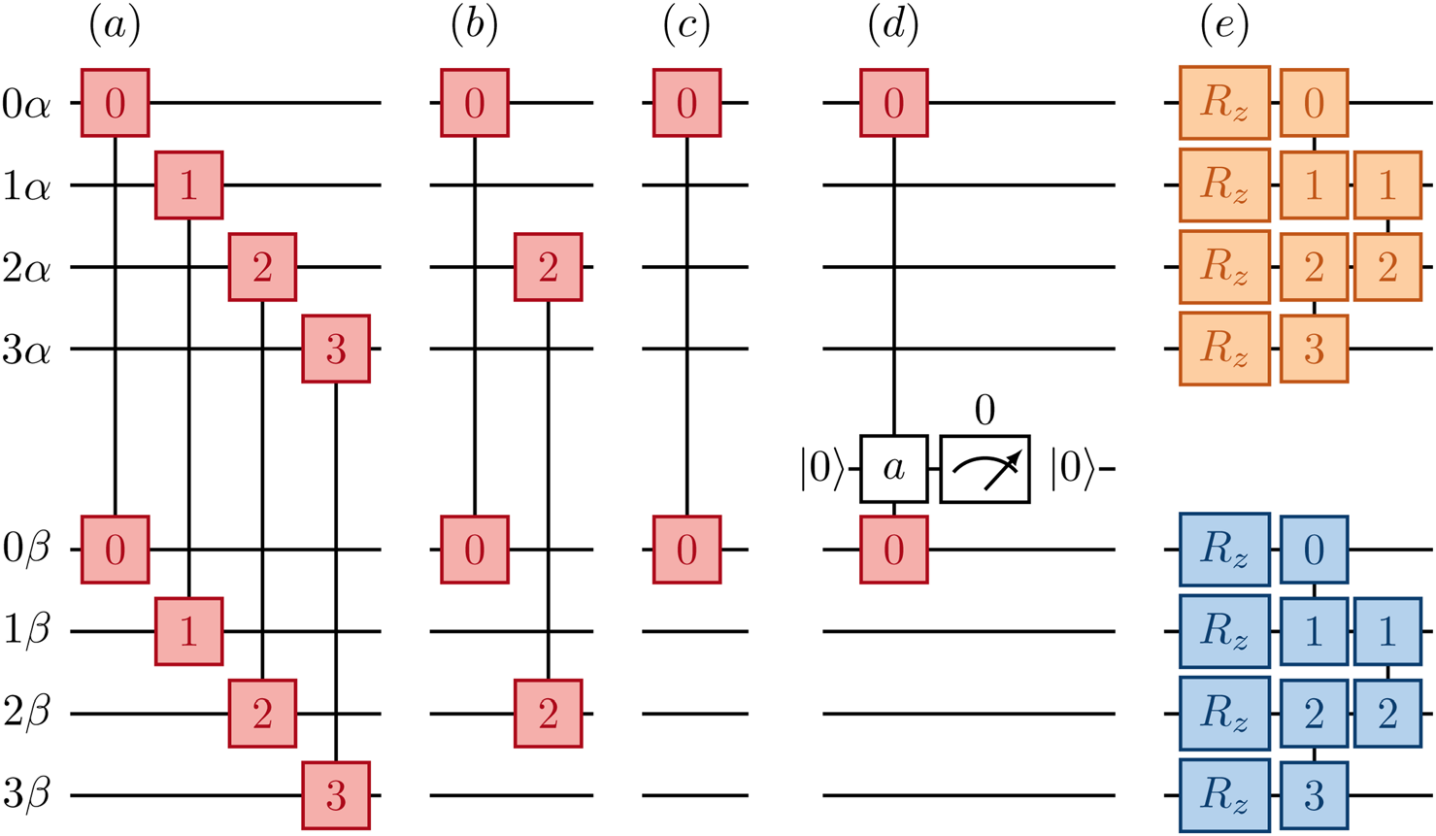
Implementation of a local cluster operator exp(*iJ*_*αα*_ + *iJ*_*ββ*_ + *iJ*_*αβ*_), illustrated for a system of *N* = 4 spatial operators. (Left) the opposite-spin operator is limited to *U*_nn_ gates acting on adjacent qubits in the topology of a square (a), hex (b), linear (c), and heavy-hex (d) lattice. For the heavy-hex lattice, the qubit interaction is generally mediated by an ancilla. (Right) (e) the same-spin operator is limited to *U*_nn_ gates compatible with linear connectivity, the same assumed in the implementation of change-of-basis unitaries.

In contrast, implementation of the LUCJ ansatzes shown in Fig. 5a–d for square, hex, and heavy-hex qubit topologies, respectively, does not require any SWAP gates. Furthermore, the opposite-spin number–number part of the LUCJ ansatz, for all qubit topologies, can be implemented in a single layer of gates, while the same-spin parts require a constant number of layers (in the present case, three). Thus, the circuit depth of the LUCJ ansatz associated with the exponential of the *Ĵ* operator no longer depends on the number of spatial orbitals, *N* (*c.f.*Fig. 5).

For the heavy-hex qubit topology, as seen in Fig. 1c, qubits associated with spin-up and spin-down orbitals (red and blue circles) are generally connected by ancillas (green circles). Correspondingly, the circuit in Fig. 5d couples two qubits (marked as 0*α* and 0*β*) with an ancilla initialized in |0〉. To implement the unitary exp(−*iφn*_0*α*_*n*_0*β*_), a gate *U*_nZn_(*φ*) = exp(−*iφn*_0*α*_*Z*_*a*_*n*_0*β*_) is applied to the three qubits. The ancilla can be measured and reset to zero, and post-selection can be carried out on the measurement outcomes to mitigate errors.

### 2.4 Connection with the Hubbard model

Jastrow wavefunctions are physically motivated ansatzes. For example, the Jastrow factor used in standard variational Monte Carlo calculations can be derived by propagating a mean-field wavefunction in imaginary time using the Feynman–Kac formula and a short-time approximation.^55,56^ The UCJ wavefunction can be derived by treating a twice-factorized low-rank decomposition of the qUCCD ansatz^22^ as a variational wavefunction.^36^ Here, we further interpret the LUCJ ansatz in terms of adiabatic state preparation (ASP).

The ASP approach starts with a Hamiltonian of a quantum system whose ground state, *Φ*_0_, is easy to prepare (*e.g.* the Fock operator *F̂*). The Hamiltonian is then slowly varied toward the target Hamiltonian, *Ĥ*, whose ground state encodes the solution to the problem. If the change is slow enough, and the Hamiltonian remains gapped along the path, the ground state of *Ĥ* can be prepared. More specifically, a quantum state |*Ψ*_*t*_〉 evolves in time according to the Schrödinger equation 
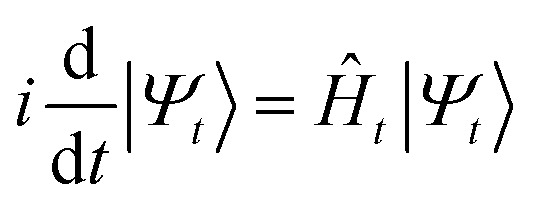
 from time *t* = 0 to time *t* = *T* starting from |*Ψ*_*t*=0_〉 = |*Φ*_0_〉. We require that *Ĥt* = 0 = *F̂* and *Ĥ*_*t*_ = *T* = *Ĥ*, which holds, *e.g.*, when 
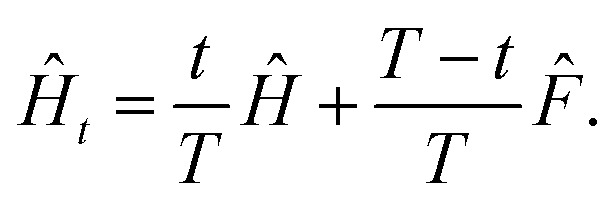
 Under suitable conditions,^15^ the state |*Ψ*_*T*_〉 converges to the ground state of *Ĥ* in the limit of *T* → ∞.

A connection between the LUCJ ansatz and ASP emerges by approximating the state |*Ψ*_*T*_〉 with a first-order Trotter formula using *M* steps of length Δ*t* = *T*/*M*,15
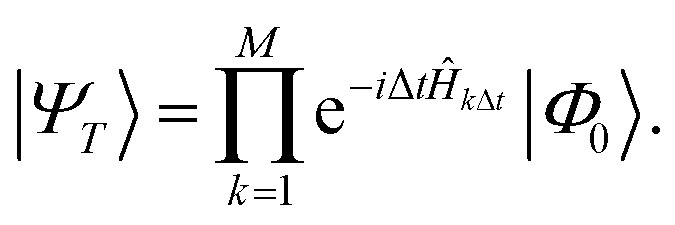


The electronic structure Hamiltonian can be expressed as16
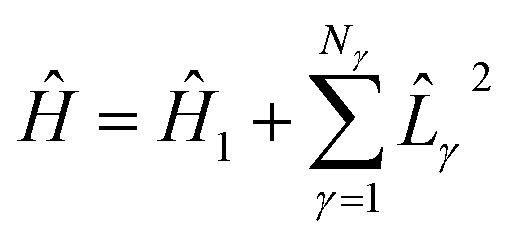
*i.e.*, the sum of a one-body operator and a sum of squares of Hermitian one-body operators *L̂*_*γ*_, obtained, *e.g.*, from a density fitting or Cholesky representation of the electron–electron repulsion. Approximating the exponential, e^−*i*Δ*tĤ*_*k*Δ*t*_^, in 15 with a first-order Trotter formula, we obtain17
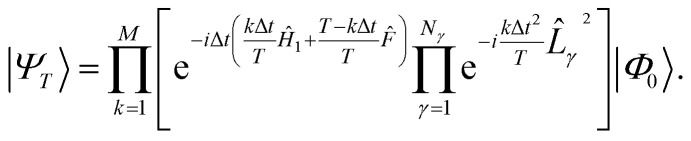


The UCJ functional form is retrieved by introducing the decomposition 
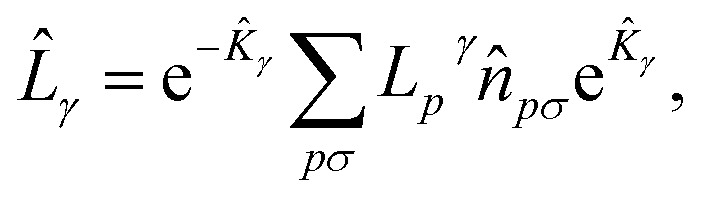
 where *L*_*p*_^*γ*^ denotes the eigenvalues of the operator *L̂*_*γ*_, and e^*K̂*_*γ*_^ implements a change of basis from the molecular-orbital basis to the eigenbasis of *L̂*_*γ*_. The eigendecomposition of *L̂*_*γ*_ leads to a factorized form for the *J* tensors, 
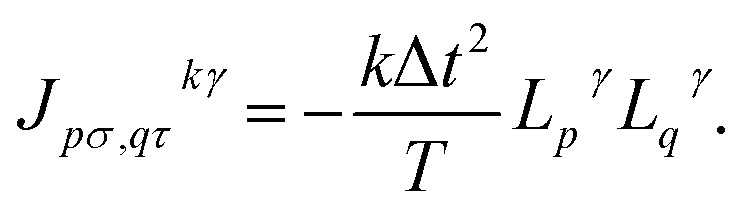
 It is known^57,58^ that the operators *L̂*_*γ*_ have low rank, *i.e.* the eigenvalues *L*_*p*_^*γ*^ of *L̂*_*γ*_ decay rapidly. In the extreme case of a rank-1 operator, one has18
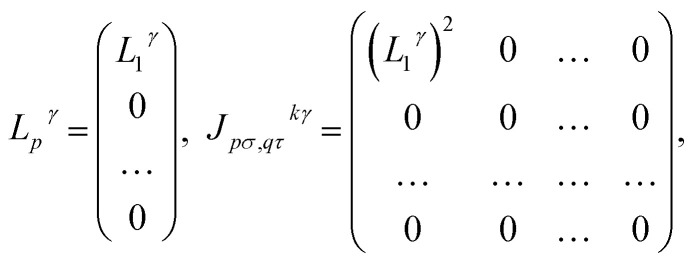
which is a local UCJ form (*i.e.*, the *J*_*pσ*,*qτ*_^*kγ*^ only has a non-zero element corresponding to *p* = *q* = 0) with *L* = *MN*_*γ*_ terms (*i.e.* one for each of the *MN*_*γ*_ matrices *J*).

Finally, we remark that eqn (17) contains the exponential of a one-body operator (a linear combination of *Ĥ*_1_ and *F̂*), which is absent in the UCJ ansatz eqn (3). Indeed, the UCJ ansatz derives from a twice-factorized low-rank decomposition of an operator comprising double excitations only, making it akin to classical CCD (coupled-cluster doubles). Like CCD, LUCJ is not stationary under orbital rotations in the active space, which are described by the exponential of a one-body operator. Therefore, to ensure stationarity under orbital rotations, and in accordance with eqn (17), we append the exponential e^*X̂*^ of a one-body orbital optimization operator *X* to the LUCJ ansatz. We also remark that recent work^59^ has indicated that various quantum computing ansatzes are not stationary under orbital rotations, and that their flexibility and accuracy improves upon addition of an orbital optimization.

In summary, ASP is designed to find the ground state of a Hamiltonian *Ĥ*, and will do so if the simulation time *T* is long enough. A Trotter approximation of ASP consists of an alternation of operators with the same functional form as the UCJ (and LUCJ assuming low-rank operators) ansatz. This observation provides a connection between ASP and LUCJ, and a further physical interpretation of this ansatz, along with the twice-factorized low-rank decomposition of the qUCCD ansatz.^22,36^

### 2.5 Computational details

All correlated calculations use the STO-6G minimal basis set. qUCCSD calculations were performed with the Qiskit program (version 0.39.0, and Qiskit nature version 0.4.0),^60^ with restricted orbitals. LUCJ calculations were performed with an in-house classical implementation (detailed in the ESI†) using the BFGS optimizer (SciPy) and PySCF version 1.7.6.^61,62^ All simulations in this work are carried out *via* linear algebra operations which do not consider the effects of shot noise or decoherence.

The bond-stretching coordinate of ethene, preserving a planar nuclear configuration (*D*_2h_ point group), was obtained from a series of constrained geometry optimizations, performed with Q-Chem^63^ version 5.4.2. These optimizations used the ωB97X-V functional^64^ and the def2-QZVPP basis.^65,66^ To consistently choose the four active orbitals along the dissociation curve, at each geometry we generated unrestricted MP2 natural orbitals and selected those with a large overlap with the target atomic orbitals, *i.e.* carbon 2p_*z*_ and sp^2^ orbitals.

Unless otherwise specified, when active spaces of canonical molecular orbitals were used, orbitals of increasing (*i.e.*, less negative) orbital energy were assigned to qubits of increasing index. When unrestricted MP2 natural orbitals were correlated, orbitals of decreasing occupation number were assigned to qubits of increasing index. The orbital to qubit assignments for cyclobutadiene, stretched ethene, and benzene are shown explicitly in Fig. S4† for each topology, corresponding to the qubit indices shown in Fig. 1.

The variational optimization of LUCJ parameters is a rather delicate operation. For instance, initializing the LUCJ ansatz with *K*_*μ*_ = *J*_*μ*_ = 0 leads to a zero-valued energy gradient vector. Indeed, expanding the unitary operator (3) to first order in *K*_*μ*_, *J*_*μ*_ leads to19
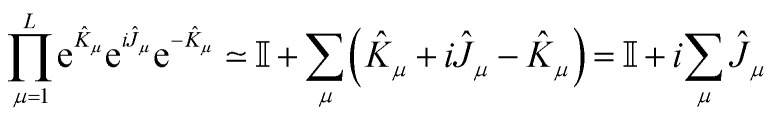
and for the energy,20
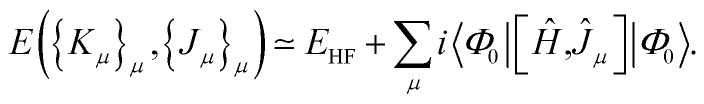


One can immediately see that the gradient with respect to *K*_*μ*_ is zero. Furthermore, when *Φ*_0_ is the HF state, the operators *Ĵ*_*μ*_ preserve it, *i.e. Ĵ*_*μ*_|*Φ*_0_〉 = *j*_*μ*_|*Φ*_0_〉. In this situation,

21 〈*Φ*_0_|[*Ĥ*,*Ĵ*_*μ*_]|*Φ*_0_〉 = 0

and thus, the parameter configuration *K*_*μ*_ = *J*_*μ*_ = 0 is a stationary point for the LUCJ energy.

In light of the above, we initialized the parameters of an LUCJ/all-to-all calculation from a truncated doubly factorized low-rank decomposition of CCSD or MP2 *t*_2_ amplitudes. We then used a “bootstrapping” procedure, employing the converged LUCJ/all-to-all, LUCJ/square, and LUCJ/hex parameters as initial guesses for LUCJ/square, LUCJ/hex, and LUCJ/heavy-hex calculations, respectively. For single-point calculations, when initializing *e.g.* a LUCJ/hex calculation from parameters of a converged LUCJ/square calculation, we permuted orbitals so that *p* ∈ *S* corresponded to the largest values of |*J*_*pp*,*αβ*_^*μ*^| (see the ESI†). The LUCJ energy must decrease monotonically with *L* for a fixed connectivity and decrease monotonically with connectivity for a fixed *L*. We reinitialized calculations from parameters pertaining to different values of *L* and/or connectivity until we no longer observed deviations from this behavior.

## 3 Results

### 3.1 Single-point calculations for square cyclobutadiene, stretched ethene, and benzene

**Fig. 6:**
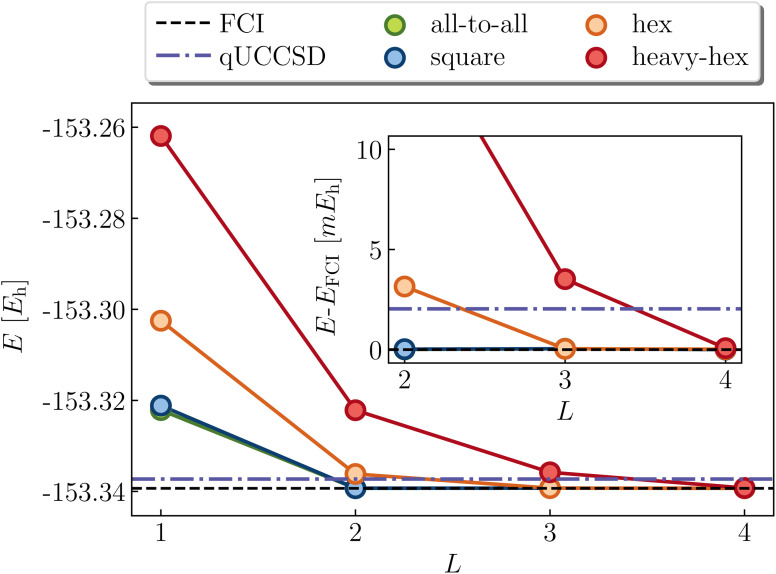
LUCJ (colored circles), qUCCSD (purple dot-dashed line) and FCI (black dashed line) ground-state energy for cyclobutadiene in a (4e,4o) active space. The SCF and qUCCSD energies are −153.169094 and −153.337275*E*_h_, respectively.

Cyclobutadiene is a highly strained, anti-aromatic molecule which exhibits a particularly challenging open-shell singlet ground-state at square geometries.^67^ We investigate a transition state geometry corresponding to the rectangular to square coordinate,^68^ wherein four carbon atoms form a square with a side length of 1.456 angstrom, with carbon–hydrogen bond lengths of 1.069 angstrom. We treat only the π-space, which consists of 4 electrons in 4 orbitals. While RHF is qualitatively wrong (predicts a triplet ground-state) and qUCCSD is beyond our target accuracy of 1.6 mHa *vs.* the exact total energy, Fig. 6 shows that all of the versions of the LUCJ ansatz can recover the exact energy. Specifically, this is accomplished by two layers (*L* = 2) for all-to-all and square approximations, by three layers for the hex ansatz, and by four layers for the heavy-hex ansatz. We note that double factorization of the qUCCSD *t*_2_ amplitudes implies *L* = 8, which enables us to quantify the resource savings for each species (*e.g.* factors of 4 fewer layers for all-to-all and square). Taken together, we find that our LUCJ approach gives better accuracy than qUCCSD, and does so with lower circuit depths. We do not expect *L* = 1 to yield meaningful results, given that the singular value decomposition of qUCCSD amplitudes implies two layers for every singular value. In what follows, we focus on *L* ≥ 2.

**Fig. 7:**
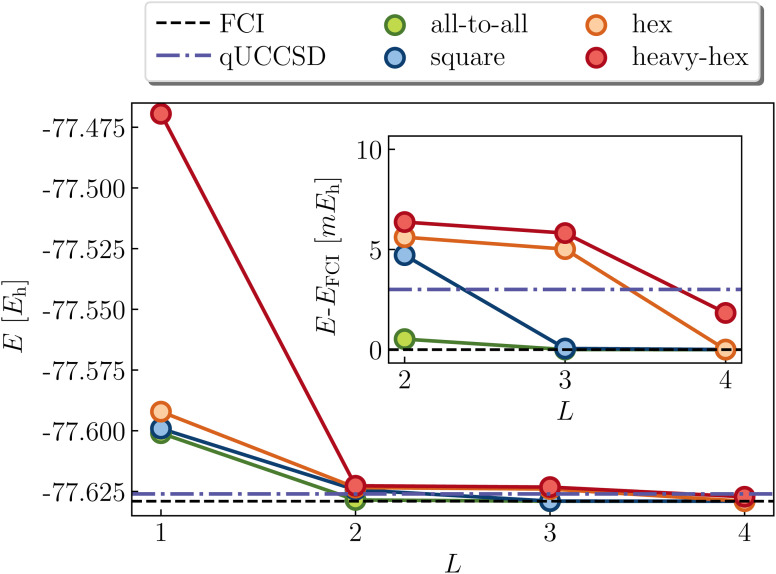
LUCJ (colored circles), qUCCSD (purple dot-dashed line) and FCI (black dashed line) ground-state energy for stretched ethene in a (4e,4o) active space. The SCF and qUCCSD energies are −77.445626 and −77.626017*E*_h_, respectively.

Another model (4e,4o) problem which exhibits strong correlation is stretched ethene. At dissociation, each methylene has two non-bonding orbitals of p and sp^2^ characters. As two well-separated methylenes are brought together to form ethene at its equilibrium geometry, these four orbitals form two bonding molecular orbitals of σ and π characters, along with the corresponding antibonding orbitals. Fig. 7 shows various calculations in an intermediate region of the double-bond breaking coordinate, at *R* = 2.0 angstroms. Unrestricted qUCCSD is shown in the dotted line, deviating from the exact energy by roughly 3 mHa. The all-to-all, square, and hex LUCJ models are converged to within 1.6 mHa of FCI by an *L* of 2, 3, and 4, respectively. The accuracy of the heavy-hex LUCJ model exceeds that of qUCCSD by *L* = 4. As was the case for square cyclobutadiene, the twice-factorized qUCCSD ansatz requires an *L* of 8, thereby requiring significantly more quantum resources to implement on the device.

**Fig. 8:**
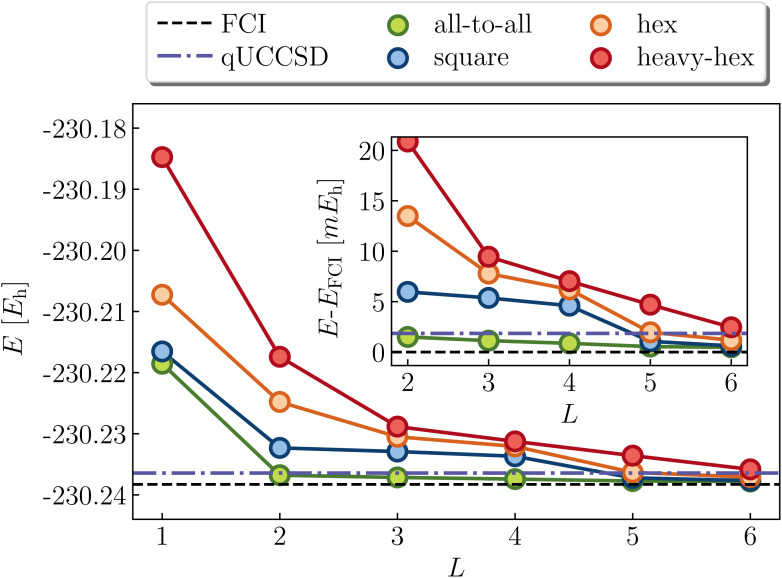
LUCJ (colored circles), qUCCSD (purple dot-dashed line) and FCI (black dashed line) ground-state energy for benzene in a (6e,6o) active space. The SCF and qUCCSD energies are −230.130155 and −230.236428*E*_h_, respectively.

Next we turn to the benzene molecule at equilibrium, and consider the (6e,6o) π space representation. Unlike square cyclobutadiene and stretched ethene, this system does not exhibit strong static correlation in the ground state. Indeed, the molecule possesses exceptional chemical stability due to its aromaticity (delocalized π system with 4*k* + 2 electrons, *k* = 1), and the wavefunction is qualitatively well-described by the RHF state. Nevertheless, there is substantial dynamic correlation (*e.g.*, the deviation of MP2 from the exact FCI energy in the active space is roughly 31 kcal mol^−1^). Fig. 8 shows the predictions of the LUCJ models alongside qUCCSD. Due to the absence of multireference characters, qUCCSD, as expected, gets rather close to the exact value, although classical CCSD is notably more accurate with a deviation of 0.2 kcal mol^−1^ (the difference between qUCCSD and CCSD is due to approximations, *e.g.*, Trotter errors incurred by the quantum implementation of the former). We note that SVD of the qUCCSD *t*_2_ amplitudes implies *L* = 18. The all-to-all UCJ ansatz provides better accuracy than qUCCSD at and after *L* = 2; the square LUCJ model by *L* = 5. While comparable accuracy is obtained with the hex and heavy-hex LUCJ ansatzes by roughly 6 layers, these circuits are still relatively shallow compared to that of qUCCSD.

### 3.2 Potential energy curves

Accurate, single-point correlation energy predictions computed at representative geometries along a reaction coordinate optimized, *e.g.*, with density functional theory, can predict most thermochemical properties (such as bond dissociation energies, redox potentials, p*K*_a_'s, spin gaps, and reaction rates) with chemically useful accuracy. However, the ability to produce smooth potential energy curves which describe both the equilibrium region and the dissociation limit is also highly desirable, *e.g.*, in simulations of chemical dynamics. In addition, pushing a chemical system at equilibrium through bond-breaking coordinates can reveal instances of strong correlation – the description of which is the motivating goal of our LUCJ ansatzes. In this section, we test the ability of LUCJ models to dissociate the single and double bonds of H_2_/LiH and ethene.

**Fig. 9:**
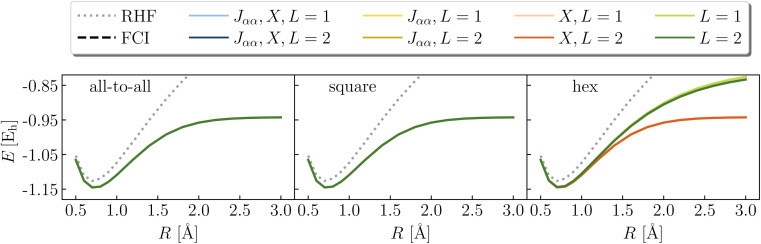
Potential energy curve of H_2_ in a (2e,2o) active space of molecular orbitals, using the LUCJ ansatz with all-to-all, square-lattice, and hex-lattice connectivities (top, middle, and bottom), with or without *J*_*αα*_ and *X* terms. All curves except those without *J*_*αα*_ and *X* terms and hex-lattice connectivity (green lines, right panels) agree with FCI within 10^−8^ hartree.

**Fig. 10:**
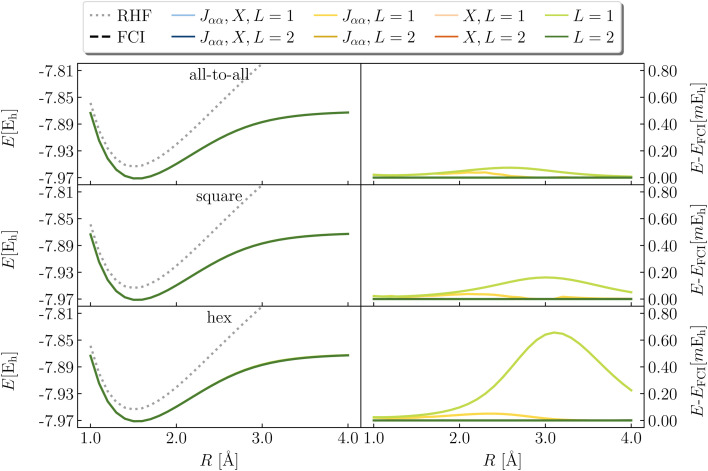
Potential energy curve of LiH in a (2e,3o) active space of UMP2 natural orbitals, using the LUCJ ansatz with all-to-all, square-lattice, and hex-lattice connectivities (top, middle, and bottom), with or without *J*_*αα*_ and *X* terms.

Fig. 9 illustrates the dissociation of H_2_ in a minimal basis (2e,2o) comparing RHF and exact values with LUCJ ansatzes with all-to-all, square, and hex qubit connectivities. As is well known, the doubly occupied RHF electron configuration cannot properly dissociate H_2_. We find that the exact dissociation curve is recovered when (1) both *J*_*αα*_ and *X*, (2) only *J*_*αα*_, and (3) only *X* are retained in the ansatz. With neither *J*_*αα*_ nor *X* (for *L* = 1,2), the potential energy curve of LUCJ is between those of RHF and FCI; the errors near the dissociation limit approach 0.12 Ha, far in excess of our target accuracy of 0.001 Ha. This can be understood by noting that in the same-spin sector of the number–number interaction term, *n̂*_*p*,*σ*_*n̂*_*p*,*σ*_ = *n̂*_*p*,*σ*_. In other words, this term of the number–number operator reduces to a one-body operator, which can be represented equivalently by the *X* operator. A similar behavior is found for the dissociation of LiH in a (2e,3o) space, shown in Fig. 10. In this case, while ansatzes with *L* = 2 yield results of FCI accuracy, ansatzes with *L* = 1 are inaccurate if either the *J*_*αα*_ or the *X* term is missing.

Now we examine the breaking of the carbon–carbon double-bond in ethene (C_2_H_4_). At equilibrium, ethene is a closed-shell singlet; at dissociation, two methylene (CH_2_) molecules each have triplet ground state multiplicity. We limit our calculations to a (4e,4o) space, keeping only the orbitals deriving from the non-bonding molecular orbitals of isolated, triplet methylene – namely 2p_*z*_ and sp^2^. Our geometry optimizations enforced planar symmetry (*D*_2h_ point group) along the dissociation. More details can be found in Section 2.5.

**Fig. 11:**
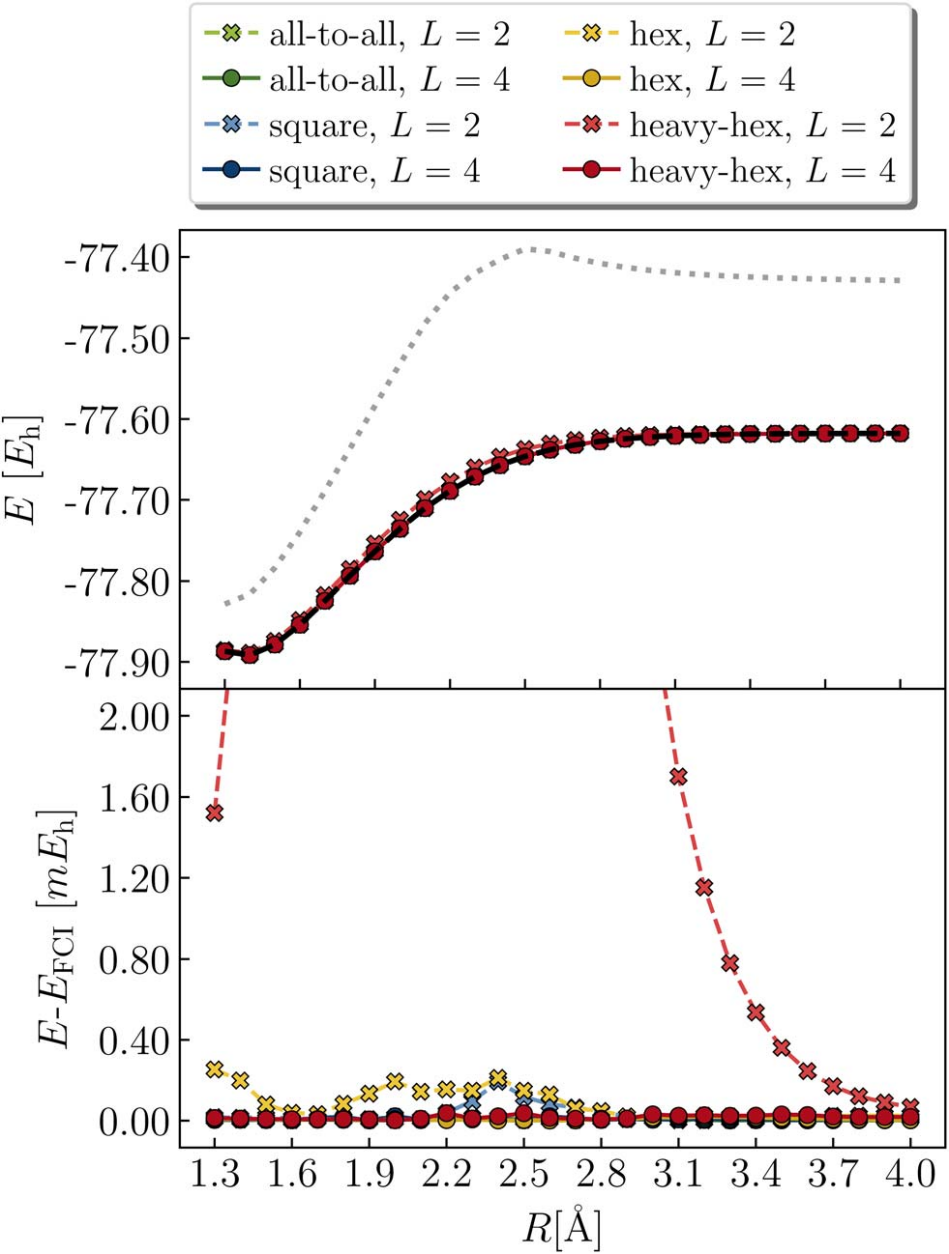
Potential energy curve of ethene in a (4e,4o) active space, using the LUCJ ansatz with all-to-all, square-lattice, hex-lattice and heavy-hex lattice (green, blue, orange, and red markers) connectivity and *L* = 2, 4 (crosses and circles, respectively).

Data from RHF and a variety of LUCJ ansatzes are compared with those of FCI as shown in Fig. 11 (results from even values of *L* are shown in the ESI†). Note that the RHF curve is not monotonic (with a “hump” between *R* = 2.3–2.5 Å) due to our choice to constrain the geometries to a plane. The LUCJ ansatzes corresponding to all-to-all, square, hex, and heavy-hex connectivities can produce exact energies at *L* > 1, 2, 3, and 4, respectively. Encouragingly, all-to-all, square, and hex LUCJ ansatzes achieve our target accuracy with *L* ≥ 2; the heavy-hex LUCJ ansatz with *L* ≥ 4. All of these LUCJ models require quantum circuits that are significantly shallower than qUCCSD.

A close inspection of the deviations of the LUCJ energy curves from exact values (Fig. 11, bottom) reveals small but notable cusps in the cases of square and hex ansatzes with *L* = 2. Albeit relatively mild (on the order of 0.1 mHa), we note that this issue has been previously reported for hardware-efficient ansatzes,^69^ and could complicate calculations of interatomic forces and vibrational properties. It is likely that these non-monotonicities are related to unsuccessful parameter optimization, as discussed further in Section 3.4.

### 3.3 Hardware perspective

The implementation of quantum algorithms on NISQ hardware faces a number of challenges. The physical time to execute an ansatz circuit must be considerably less than the qubit coherence times, *T*_1_ and *T*_2_, which currently are around 100 μs on state-of-the-art superconducting devices. By contrast, the duration of a two-qubit gate is around 400 ns, so that around 250 layers of two-qubit gates can be executed before qubit decoherence. A second, and arguably more stringent, challenge arises due to various types of errors that result from applying any quantum operation, *e.g.* a 2 qubit gate. As a simple numerical example, even with 2 qubit gate fidelities of 99%, the application of 11 2 qubit gates will lead to fidelities of <90%.

Localization of the previously proposed UCJ ansatz enables a number of significant advantages from the perspective of quantum resources required to encode the state. Gate counts in terms of the number of spatial orbitals are shown in Table 1. All flavors of the LUCJ approximation do not require SWAP gates. Furthermore, with regard to the e^*iĴ*^ part, the circuit depth no longer depends on *N*. Another key point is that the circuit depth for the square, hex, and linear connectivities is the same – namely, 4 (three from the same-spin part and one from the opposite-spin part). Finally, the required number of number–number gates, *U*_nn_, is reduced from quadratic to linear in *N*. Device connectivities with fewer neighboring qubits require proportionally fewer *U*_nn_ gates.

**Table 1:** Computational cost of the operations comprising a layer of the Jastrow ansatz, for a system of electrons in *N* spatial orbitals

Instruction	*R*_*z*_	*G*	SWAP	*U*_nn_	*cX*	Depth
e^*K*^	2*N*	*N*(*N* − 1)	0	0	0	(1 + *N*)
e^*iJ*^ all-to-all	2*N*	0	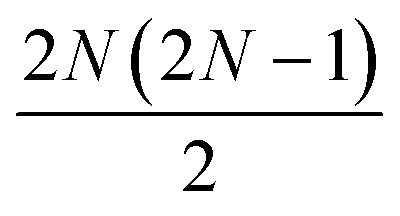	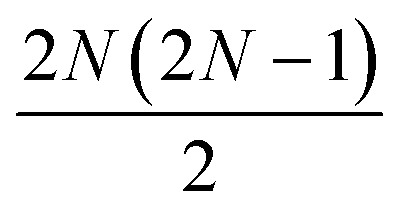	0	(1 + 4*N*)
e^*iJ*^ square	2*N*	0	0	*N* + 2(*N* − 1)	0	4
e^*iJ*^ hex	2*N*	0	0	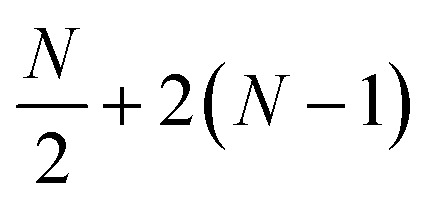	0	4
e^*iJ*^ heavy-hex	2*N*	0	0	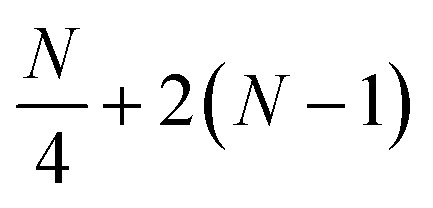	*N*	8
e^*iJ*^ linear	2*N*	0	0	1 + 2(*N* − 1)	0	4

In the case of a heavy-hex topology, as indicated in Fig. 5, 4 CNOT gates are required to implement an *R*_*zzz*_ gate in the presence of an ancilla. This operation has to be repeated *N*/4 times since the ratio between ancillas and orbitals, as illustrated in Fig. 2, is 4. Therefore, we have a total of *N* CNOT gates.

**Fig. 12:**
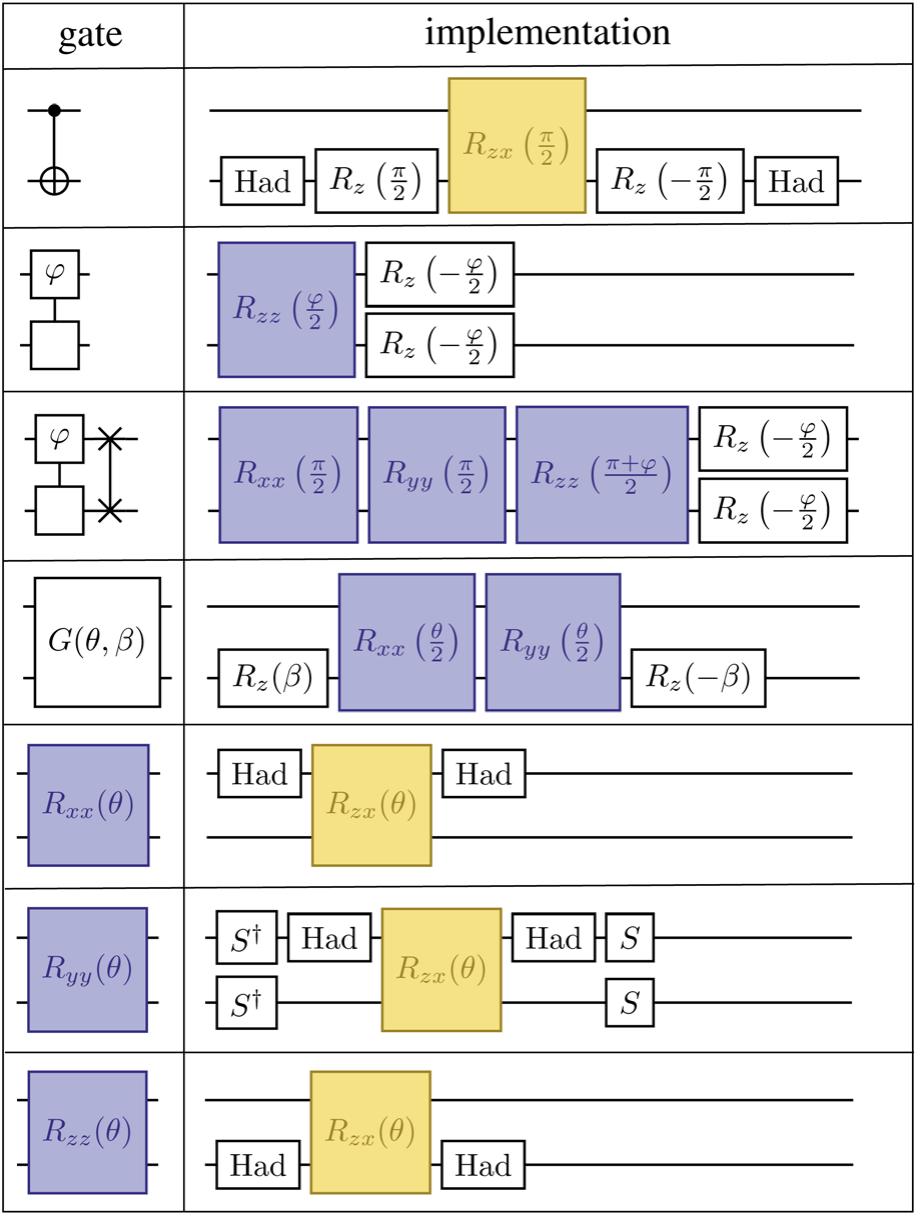
Implementation of CNOT, *U*_nn_, SWAP·*U*_nn_, and XX + *YY* gates (rows 1–4 from above) with single- and two-qubit gates. The *R*_*xx*_, *R*_*yy*_, and *R*_*zz*_ gates (rows 5–7) along with the CNOT gate can be implemented with single-qubit gates and the *R*_*zx*_ gate (yellow block), native to superconducting devices with fixed-frequency qubits.

In addition, another salient feature of the LUCJ ansatzes is natural and efficient implementation on superconducting quantum hardware with tunable couplers. Fig. 12 shows the decomposition of the *U*_nn_ gates and Givens rotations into sequences of single-qubit gates along with *R*_*xx*_, *R*_*yy*_, and *R*_*zz*_ 2 qubit gates (bottom three rows). These *R* gates are then further decomposed into native 2 qubit gates, *R*_*zx*_, highlighted in yellow, which can be implemented in ∼400 ns. On such fixed-frequency devices, each number–number gate requires an *R*_*zx*_ native gate, while each Givens rotation requires two native gates. Furthermore, the all-to-all UCJ model requires many *U*_nn_·SWAP gates, each of which requires 3 native gates.

**Fig. 13:**
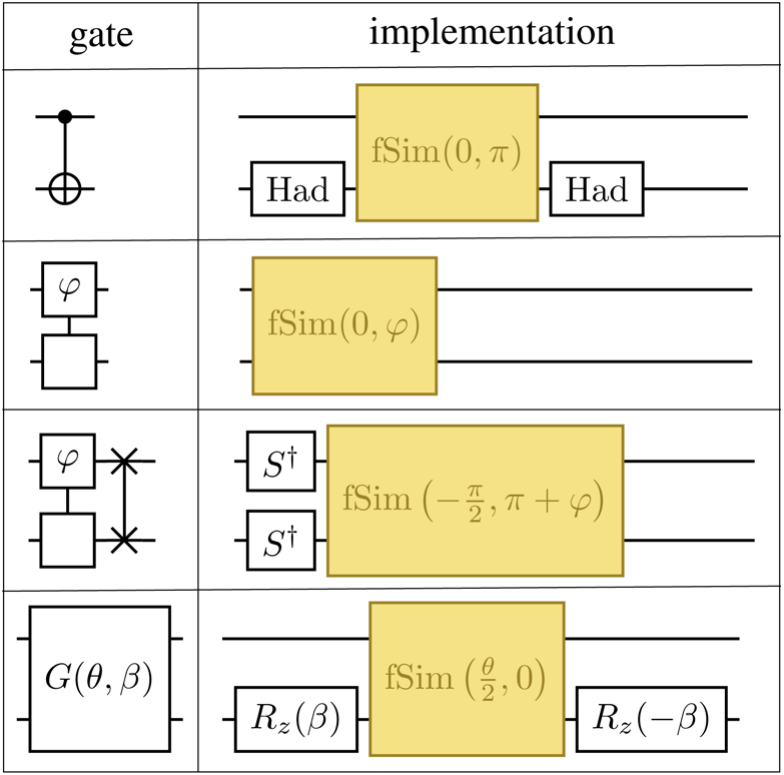
Implementation of CNOT, *U*_nn_, *U*_nn_·SWAP, and XX + *YY* gates (top to bottom) with single-qubit gates and the two-qubit fSim(*θ*, *ϕ*) gate (yellow block), native to superconducting devices with tunable couplers.

In contrast, Fig. 13 shows the decomposition of the 2 qubit gates required in the LUCJ ansatzes onto the native gates on devices with tunable couplers,^49,70^ which is denoted as fSim and is represented by the matrix22
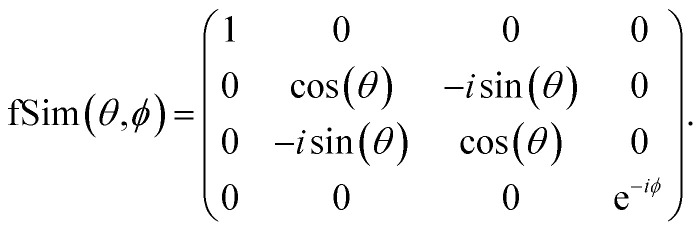


The *U*_nn_ gate is itself a native gate, and can be implemented with 30 ns gate times. This is an order of magnitude faster than is possible on fixed-frequency devices. A Givens rotation can be implemented with a single fSim native gate. When accounting for the 10× speed-up for each native gate, the total gate-time for a Givens rotation is 20× faster when utilizing devices with tunable couplers.

### 3.4 Parameter optimization

**Fig. 14:**
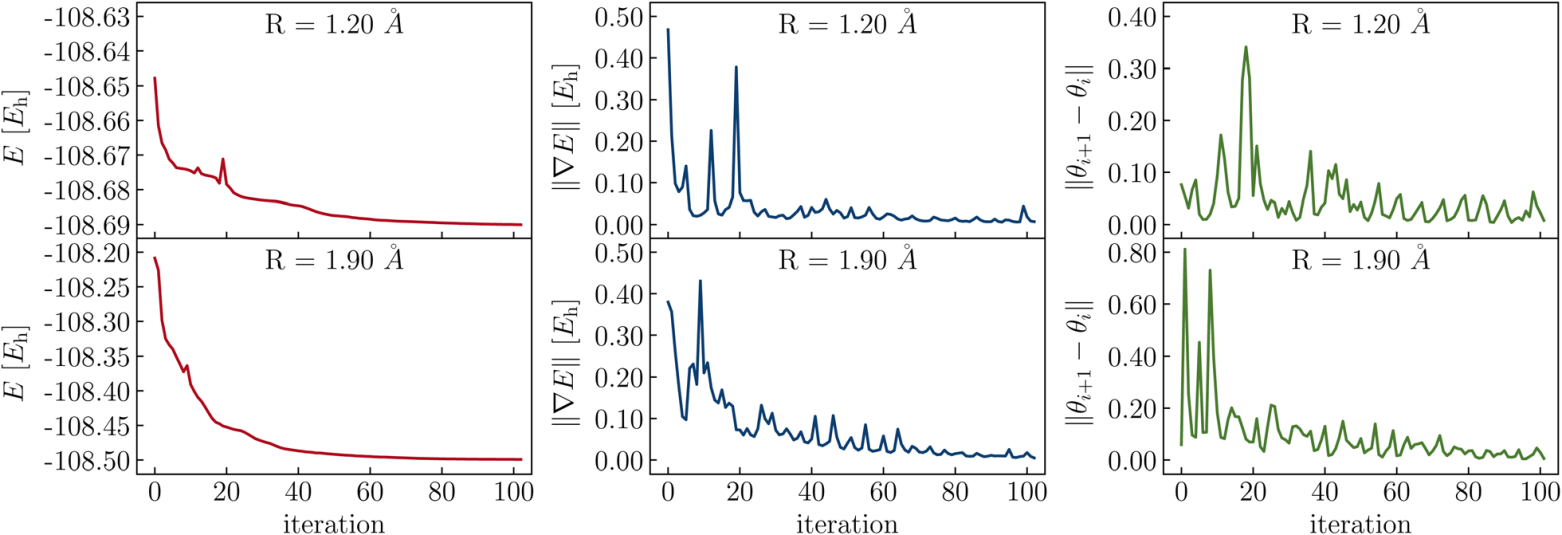
Optimization of the LUCJ ansatz for N_2_ in a (6e,6o) active space of UMP2 natural orbitals at bond lengths *R* = 1.20 and 1.90 Å (top and bottom), using the LUCJ ansatz with square-lattice connectivity and *L* = 4. Left, middle, and right panels show the energy, energy gradient, and parameter variations (red, blue, and green, respectively) as the optimization proceeds.

The parameters in the LUCJ ansatzes are optimized variationally. For the majority of the systems investigated in this work, parameter optimization with the BFGS algorithm has not presented insurmountable difficulties, owing to the bootstrapping and parameter initialization protocols developed and employed. However, in Fig. 14 we show a difficult case, namely the optimization of LUCJ parameters for the N_2_ molecule near equilibrium (*R* = 1.2 Å) and near dissociation (*R* = 1.9 Å). In both cases, the optimization procedure features cusps in the energy and pronounced oscillations both in the norm of the gradient and in the distance between variational parameters in consecutive iterations.

Future work will involve, first and foremost, accelerating and increasing the robustness of the parameter optimization scheme. Second-order solvers which make use of the exact Hessian will be explored. Given the success of classical variational quantum Monte Carlo, a promising step forward will be to optimize the LUCJ parameters *via* the linear method.^71–73^ Another complementary strategy involves further reducing the number of parameters in the LUCJ ansatzes. This could be done, *e.g.*, by discarding all same-spin *J*_*σσ*_ terms, and offloading the corresponding correlation energy contributions *via* polynomially scaling classical algorithms. This appears plausible given that *U*_nn_-type correlations are predominately from opposite-spin electrons which can occupy the same orbital (same-spin correlation is longer-ranged due to the Pauli principle).

## 4 Conclusions and outlook

In this work we have presented a new ansatz – the localized unitary cluster Jastrow (LUCJ) wavefunction – which can describe both dynamic and static electron correlations accurately and is amenable to efficient implementation on superconducting quantum devices. While the computational cost of exactly measuring the energy is super-polynomial on classical devices, it is polynomial on quantum devices. We have drawn inspiration from the repulsive Hubbard model, which penalizes double-occupancy of lattice sites, and built this physics into a correlator which acts on a closed-shell, mean-field (RHF) state. The LUCJ ansatz is flexible enough to include the qUCCSD wavefunction, yet retains an avenue (*via* increasing the number of layers, *L*) to systematically approach the exact state, even in the presence of multi-reference characters. While we focus on the LUCJ model defined by a square configuration of qubits without SWAP gates – *i.e.*, keeping only on-site opposite-spin and nearest-neighbor same-spin number–number interactions – we also demonstrate that with more sparse arrangements of qubits, *e.g.* the hex and heavy-hex topologies, one can also converge to very high accuracy with the addition of more layers. In this work we have investigated an antiaromatic compound (square cyclobutadiene) and strongly correlated regimes encountered when stretching single, double, and triple bonds. We are encouraged that compact and hardware-friendly LUCJ ansatzes led to the appropriate dissociation limits, which is challenging to achieve even with sophisticated coupled cluster methods.^74–76^ We also made resource estimates which quantify substantial reductions in gates and gate-times, in particular for devices with tunable couplers.

We envision that the LUCJ ansatz will not only be a leading way to perform variational quantum simulations,^6^ but will be an advantageous choice to initialize quantum subspace expansion (QSE), imaginary-time evolution, adiabatic state preparation, and quantum phase estimation calculations in a fault-tolerant era. Developing more robust approaches for parameter optimization will enable us to investigate larger and more complex systems, and we are eager to test whether such a compact ansatz (small values of *L*) will continue to preserve near-exact accuracy. Future directions include exploring this variationally optimized LUCJ decomposition in the context of time propagation, and as a single-reference ansatz form for the recently proposed non-orthogonal quantum eigensolver approach.^38^ In light of the LUCJ ansatzes' ability to capture strong correlations with minimal quantum resources, we will explore its use as an active space solver in the contexts of tailored and externally corrected coupled-cluster schemes.^77–81^ In this work we have elected to investigate the LUCJ ansatz in the absence of shot noise and decoherence; future work will involve its characterization in more realistic simulations and on quantum hardware.

As a final comment, the research community to date has largely been pursuing electronic structure ansatzes that are either motivated completely by hardware efficiency or by physical intuition. The family of LUCJ ansatzes introduced herein demonstrates that a useful balance between the two priorities is possible, *i.e.* the dominant type of electron correlation in many strongly correlated systems (involving opposite-spin electrons in a spatial orbital) is faithfully retained without requiring SWAP operations which are a leading bottleneck in terms of reducing circuit depth. It is our hope that this hybrid design principle for quantum chemical ansatzes will inspire future developments in the field.

## Data availability

The data that support the findings of this study are available from the corresponding authors upon reasonable request.

## Author contributions

J. S. and M. M. conceived the project. M. M., K. S., and J. S. developed the quantum simulation codebase. M. M. carried out numerical simulations. J. S., M. M., M. H. G., and K. B. W. analyzed computational results. All authors participated in the composition and revision of the manuscript.

## Conflicts of interest

There are no conflicts of interest to declare.

## Supplementary Material

SC-014-D3SC02516K-s001
