# Supplementary material for: Bridging physical intuition and hardware efficiency for correlated electronic states: the local unitary cluster Jastrow ansatz for electronic structure
Source: Chem Sci. 2023 Sep 21;14(40):11213–27. doi: 10.1039/d3sc02516k (PMC10583744; doi:10.1039/d3sc02516k)
Supplement: SC-014-D3SC02516K-s001 [file SC-014-D3SC02516K-s001.pdf]

Electronic Supplementary Material (ESI) for Chemical Science.
This journal is © The Royal Society of Chemistry 2023

Supplemental Material for "Bridging physical intuition and hardware efficiency for correlated electronic states: the local unitary cluster Jastrow ansatz for electronic structure"

Mario Motta,[1,*] Kevin J. Sung,[2] K. Birgitta Whaley,[3,4,5] Martin Head-Gordon,[3,6] and James Shee[3,7,†]

[1] IBM Quantum, IBM Research – Almaden, San Jose, CA 95120, USA
[2] IBM Quantum, IBM T.J. Watson Research Center, Yorktown Heights, NY 10598, USA
[3] Department of Chemistry, University of California, Berkeley, CA 94720, USA
[4] Berkeley Quantum Information and Computation Center, University of California, Berkeley, CA 94720, USA
[5] Challenge Institute for Quantum Computation, University of California, Berkeley, CA 94720, USA
[6] Chemical Sciences Division, Lawrence Berkeley National Laboratory, Berkeley, CA 94720, USA
[7] Department of Chemistry, Rice University, Houston, TX 77005, USA

S1. UNITARY COUPLED CLUSTER CALCULATIONS

In Table S1, we report additional calculations with the unitary coupled cluster ansatz (qUCCSD). In the case of ethene, unrestriction is necessary to approach the FCI energy $E = -77.629025$ Hartree, although it produces a spin-contaminated state with $\langle S^2 \rangle \simeq 0.98$. In the cases of cyclobutadiene and benzene, we observe no spin contamination, and good agreement between all the implementations of qUCCSD and with the FCI energies of $E = -153.339314$ and $E = -230.238284148$ Hartree, respectively.

molecule	flavor	formula	ordering	energy [E_h]
C_2H_4	R	Trotter	d+s	-77.529519
C_2H_4	R	Trotter	s+d	-77.529519
C_2H_4	R	Suzuki	d+s	-77.529521
C_2H_4	R	Suzuki	s+d	-77.523434
C_2H_4	U	Trotter	d+s	-77.626017
C_2H_4	U	Trotter	s+d	-77.626017
C_2H_4	U	Suzuki	d+s	-77.626017
C_2H_4	U	Suzuki	s+d	-77.626017
C_4H_4	R	Trotter	d+s	-153.337269
C_4H_4	R	Trotter	s+d	-153.337274
C_4H_4	R	Suzuki	d+s	-153.337274
C_4H_4	R	Suzuki	s+d	-153.337275
C_4H_4	U	Trotter	d+s	-153.337275
C_4H_4	U	Trotter	s+d	-153.337275
C_4H_4	U	Suzuki	d+s	-153.337275
C_4H_4	U	Suzuki	s+d	-153.337275
C_6H_6	R	Trotter	d+s	-230.236417
C_6H_6	R	Trotter	s+d	-230.236396
C_6H_6	R	Suzuki	d+s	-230.236417
C_6H_6	R	Suzuki	s+d	-230.236396
C_6H_6	U	Trotter	d+s	-230.236431
C_6H_6	U	Trotter	s+d	-230.236430
C_6H_6	U	Suzuki	d+s	-230.236431
C_6H_6	U	Suzuki	s+d	-230.236430

TABLE S1. qUCCSD calculations carried out in this study. "flavor" refers to a restricted closed-shell (R) or unrestricted (U) implementation. "formula" refers to whether the exponential of the cluster operator is approximated with a Trotter or Suzuki decomposition. "ordering" refers to the ordering of operators (exponentials of doubles followed by singles, d+s, or viceversa, s+d) in the product formula approximation.

* mario.motta@ibm.com
† james.shee@rice.edu

S2. DISSOCIATION OF ETHENE

In Figure S1 we study the dissociation of ethene using the LUCJ ansatze with $L = 1, 2, 3, 4$. For $L = 1$, the accuracy of LUCJ is very limited, with all-to-all, square, and hex ansatzes overestimating the dissociation energy by 50 milliHartree, and heavy-hex by more than 100 milliHartree. The accuracy of LUCJ improves with increasing L. With the exception of heavy-hex, all ansatzes with $L = 2$ differ from FCI by less than 1.6 milliHartree.

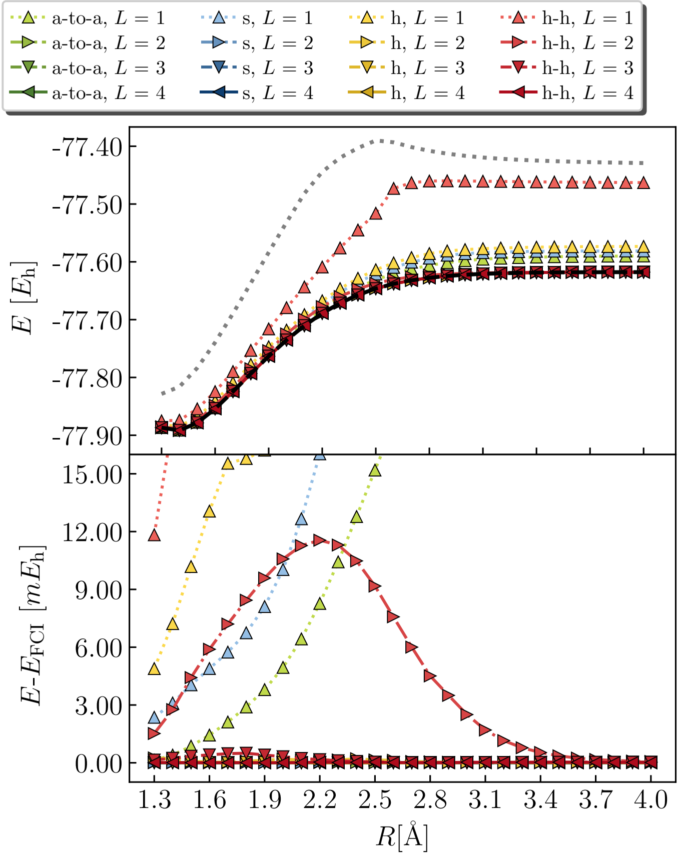

FIG. S1. Potential energy curve of ethene in a (4e,4o) active space, using the LUCJ ansatz with all-to-all, square-lattice, hex-lattice, and heavy-hex lattice (green, blue, orange, red markers denoted "a-to-a", "s", "h", and "h-h" for brevity) connectivity and $L = 1, 2, 3, 4$ (up-, right-, down-, left-pointing triangles respectively).

S3. ADDITIONAL LUCJ CALCULATIONS

In Figures S2 and S3 we report additional LUCJ calculations for the H_2 and LiH molecules, in a basis of two Lowdin orbitals and three natural orbitals in the A_1 irrep of the $C_{\infty v}$ group, respectively.

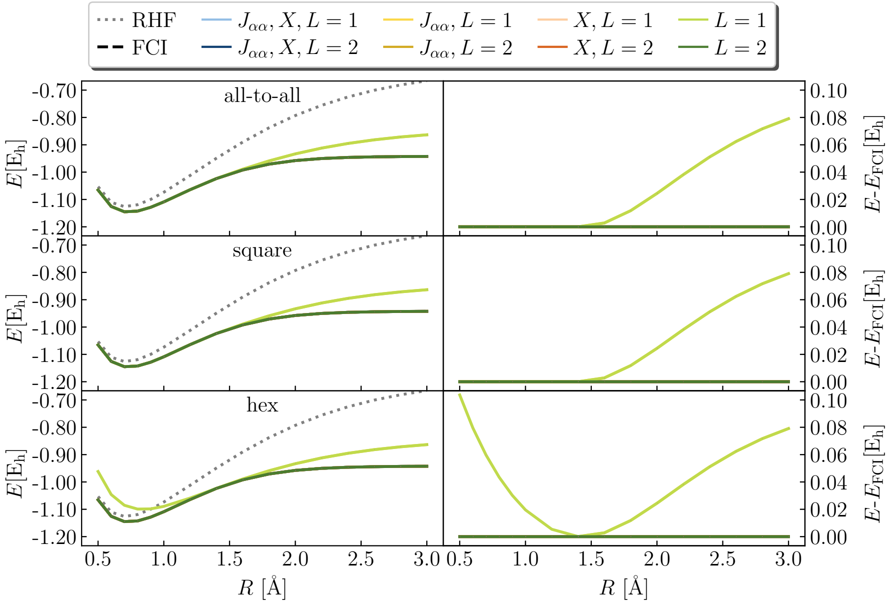

FIG. S2. Potential energy curve of H_2 in a (2e,2o) active space of Löwdin orbitals, using the LUCJ ansatz with all-to-all, square-lattice, hex-lattice connectivity (top, middle, bottom), with or without $J_{\alpha\alpha}$ and X terms.

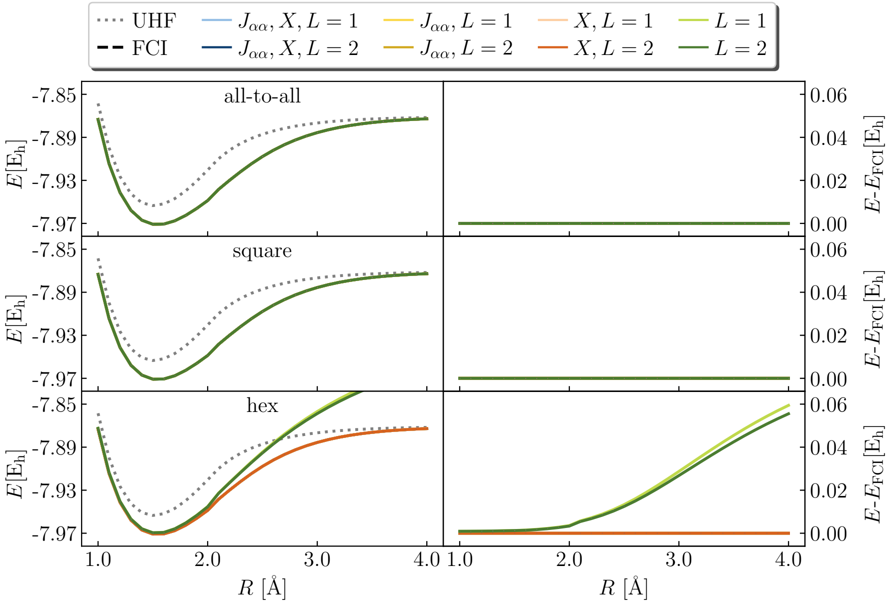

FIG. S3. Potential energy curve of LiH in a (2e,2o) active space of natural orbitals, using the LUCJ ansatz with all-to-all, square-lattice, hex-lattice connectivity (top, middle, bottom), with or without $J_{\alpha\alpha}$ and X terms. All curves except those without $J_{\alpha\alpha}$ and X terms and hex-lattice connectivity (green lines, bottom panels) agree with FCI within 10^{-6} Hartree.

S4. COMPUTATIONAL DETAILS FOR CLASSICAL SIMULATION OF THE LUCJ ANSATZ

For single-point calculations, when initializing e.g. a LUCJ/hex calculation from parameters of a converged LUCJ/square calculation, we permuted orbitals so that $p \in S$ corresponded to the largest values of $|J^{\mu}_{pp,\alpha\beta}|$. The indices of the qubits associated with the active-space orbitals of cyclobutadiene, stretched ethene, and benzene are shown in Figure S4. This choice does not affect the LUCJ energy, as orbital permutations can be subsumed in the change-of-basis operators $e^{\hat{K}_\mu}$, but may affect parameter optimization.

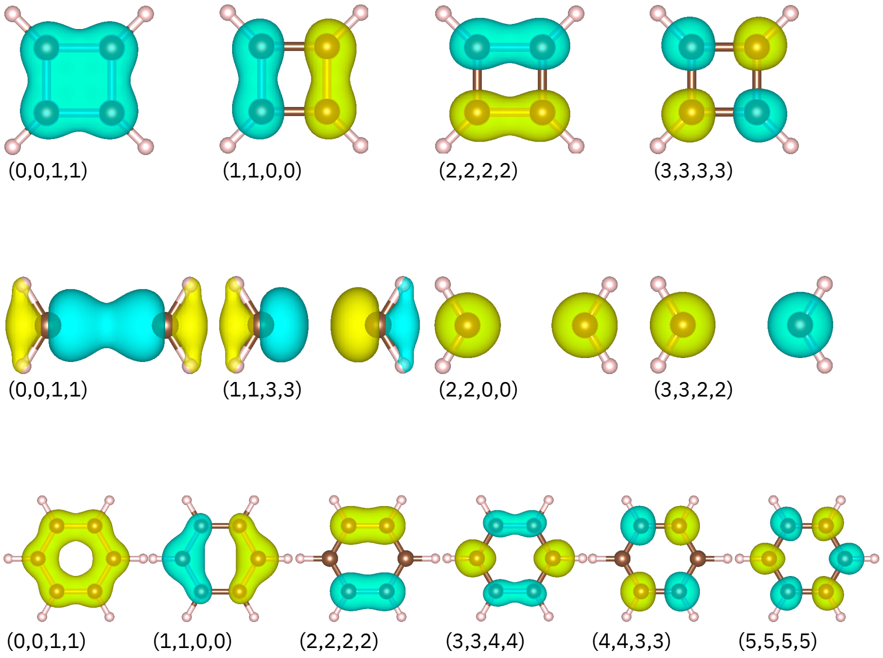

FIG. S4. Molecular orbitals for cyclobutadiene (top), stretched ethene (middle), and benzene (bottom), and indices of the qubits associated with them in LUCJ ansatzes with all-to-all, square-lattice, hexagonal-lattice and heavy-hex lattice topologies (left to right, in round brackets).

Simulating the LUCJ ansatz involves the application of the unitaries $e^{\hat{K}_\mu}$ and $e^{i\hat{J}_\mu}$ on a wavefunction, where

$$\hat{K}_\mu = \sum_{pq,\sigma} K^{\mu}_{pq}\, \hat{a}^\dagger_{p\sigma}\hat{a}_{q\sigma} \ , \ \hat{J}_\mu = \sum_{pq,\sigma\tau} J^{\mu}_{pq,\sigma\tau}\, \hat{n}_{p\sigma}\hat{n}_{q\tau} \ . \tag{S1}$$

Because these unitaries conserve particle number and the Z-component of spin, we can restrict the simulation to the subspace spanned by basis vectors with the same particle number and spin as the initial state. The dimension of this subspace is $D = \binom{N}{N_\alpha}\binom{N}{N_\beta}$ where N is the number of orbitals, N_σ is the number of particles of spin σ, and $\binom{n}{k}$ is the binomial coefficient. The basis vectors are indexed by pairs of bitstrings (I_α, I_β), where I_σ is a bitstring of length N whose j-th bit is 1 if and only if orbital j of spin σ is occupied, and we use the notation $j \in I_\sigma$ to indicate this is the case. The coefficient corresponding to the basis vector (I_α, I_β) is denoted $\gamma_{I_\alpha,I_\beta}$, or simply γ_I if we wish to ignore the spin. This wavefunction representation is the same as that used in classical full configuration-interaction (FCI) calculations, so existing FCI code can be repurposed. To this end, we used the open-source software package PySCF in our simulations [1]. To simulate the LUCJ ansatz, we considered two methods.

A. Taylor series method

The exponential action of the operators (S1) can be approximated using a Taylor series expansion. This method requires only the ability to apply the operators (S1) to a wavefunction. Because similar operations are also performed in classical FCI calculations, it was relatively straightforward to implement these operations using PySCF. The application of a single term in a sum incurs a computational cost scaling as $\mathcal{O}(DN^2)$. To achieved a fixed precision, a constant number of terms in the Taylor series needs to be included. In practice, we found that fewer than 100 terms were needed to achieve a convergence threshold of 10^{-12} as measured by the Euclidean norm of the residual vector.

B. Exact method via unitary decompositions

We also considered simulation methods inspired by quantum circuit implementations of the unitaries. These methods utilize a decomposition of the unitary into a sequence of basic operations and directly apply these operations by updating the coefficients stored in the state vector. While these methods have the same asymptotic complexity as the Taylor series method, the constant factors are much smaller, and we found that in practice they were on the order of a hundred times faster. In the following sections, we describe how to implement each of the two kinds of unitaries.

1. *Orbital rotation*

The unitary $e^{\hat{K}_\mu}$ represents a rotation of the orbital basis. It can be decomposed as

$$e^{\hat{K}_\mu} = \mathcal{P}_1 \cdots \mathcal{P}_N \mathcal{G}_1 \cdots \mathcal{G}_{N_G}, \tag{S2}$$

where each $\mathcal{P}_j$ is a phase operator of the form

$$\mathcal{P}_j = \prod_\sigma \exp\left(i\varphi_j \hat{a}^\dagger_{j\sigma} \hat{a}_{j\sigma}\right) \tag{S3}$$

and each $\mathcal{G}_j$ is a Givens rotation operator acting on neighboring fermionic modes [2, 3]. A Givens rotation operator $\mathcal{G}$ acting on modes p and q has the action

$$\begin{pmatrix} \mathcal{G}\hat{a}^\dagger_{p\sigma}\mathcal{G}^\dagger \\ \mathcal{G}\hat{a}^\dagger_{q\sigma}\mathcal{G}^\dagger \end{pmatrix} = G \begin{pmatrix} \hat{a}^\dagger_{p\sigma} \\ \hat{a}^\dagger_{q\sigma} \end{pmatrix}, \tag{S4}$$

where G is a 2×2 unitary matrix of the form

$$G = \begin{pmatrix} c & s \\ -s^* & c \end{pmatrix}. \tag{S5}$$

The number of Givens rotations is $N_G = N(N-1)/2$. The Givens rotation matrices and phase angles can be calculated from the matrix e^{K^μ} using the procedure described in Ref. [3]. Once these quantities are calculated, the orbital rotation can be achieved by applying the sequence of operations given by Eq. (S2) directly on the state vector. In the following, we will describe how to apply the operations considering a single spin. The full operation is achieved by applying the single-spin operation for both spins in sequence.

Let γ_I denote a coefficient of the original state vector and γ'_I a coefficient of the transformed state vector. Applying a phase operator $\mathcal{P}_j$ amounts to multiplying by a phase the coefficients corresponding to basis states in which the corresponding orbital is occupied:

$$\gamma'_I = \begin{cases} e^{i\varphi}\gamma_I & \text{if } j \in I \\ \gamma_I & \text{otherwise.} \end{cases} \tag{S6}$$

Applying a Givens rotation $\mathcal{G}$ acting on neighboring modes p and q is achieved by rotating the subspaces spanned by basis vectors in which exactly one of these modes is occupied:

$$\gamma'_I = \begin{cases} c\gamma_I + s\gamma_{I_{p\mapsto q}} & \text{if } p \in I \text{ and } q \notin I \\ -s^*\gamma_{I_{q\mapsto p}} + c\gamma_I & \text{if } q \in I \text{ and } p \notin I \\ \gamma_I & \text{otherwise.} \end{cases} \tag{S7}$$

Here, the notation $I_{p\mapsto q}$ denotes the bitstring obtained from I by setting bit p to 0 and bit q to 1. Since there are $\mathcal{O}(N^2)$ total operations to apply, the compute time for performing a full orbital rotation is $\mathcal{O}(DN^2)$.

2. *Diagonal Coulomb operator*

The unitary $e^{i\hat{J}_\mu}$ is equal to a product of commuting terms where each term has the form $\exp(i\varphi\hat{n}_{p\sigma}\hat{n}_{q\tau})$ for some angle φ. This term is a diagonal operator with the following action:

$$\gamma'_{I_\alpha,I_\beta} = \begin{cases} e^{i\varphi}\gamma_{I_\alpha,I_\beta} & \text{if } p \in I_\sigma \text{ and } q \in I_\tau \\ \gamma_{I_\alpha,I_\beta} & \text{otherwise.} \end{cases} \tag{S8}$$

The number of terms is $\mathcal{O}(N^2)$, so the total compute time is $\mathcal{O}(DN^2)$.

[1] Q. Sun, T. C. Berkelbach, N. S. Blunt, G. H. Booth, S. Guo, Z. Li, J. Liu, J. D. McClain, E. R. Sayfutyarova, S. Sharma, *et al.*, Pyscf: the python-based simulations of chemistry framework, WIREs Comput. Mol. Sci **8**, e1340 (2018).

[2] Z. Jiang, K. J. Sung, K. Kechedzhi, V. N. Smelyanskiy, and S. Boixo, Quantum algorithms to simulate many-body physics of correlated fermions, Phys. Rev. Appl **9**, 044036 (2018).

[3] W. R. Clements, P. C. Humphreys, B. J. Metcalf, W. S. Kolthammer, and I. A. Walmsley, Optimal design for universal multiport interferometers, Optica **3**, 1460 (2016).
